# Serology as an early diagnostic tool in pediatric patients with Shiga toxin-producing *Escherichia coli*-associated hemolytic uremic syndrome: a *post hoc* analysis of a phase 2 clinical trial

**DOI:** 10.1128/jcm.01415-25

**Published:** 2026-02-27

**Authors:** Stella M. Landivar, Luciano J. Melli, Mariana Pichel, Marta Rivas, Mariana Colonna, Carolina Massa, Vanesa Zylberman, Santiago Sanguineti, Linus Spatz, Fernando Goldbaum, Diego J. Comerci, Juan E. Ugalde, Andrés E. Ciocchini, INM004 Study Group, Alicia Fayad

**Affiliations:** 1 Instituto de Investigaciones Biotecnológicas, Universidad Nacional de San Martín (UNSAM)-Consejo Nacional de Investigaciones Científicas y Técnicas (CONICET), Escuela de Bio y Nanotecnologías (EByN), Universidad Nacional de San Martín, San Martín, Buenos Aires, Argentina; 2 Inmunova S. A. 738969 , San Martín, Argentina; Vanderbilt University Medical Center, Nashville, Tennessee, USA

**Keywords:** hemolytic uremic syndrome, STEC, phase 2 clinical trial, diagnostic, serodiagnosis, glycoprotein, ELISA, LFIA

## Abstract

The specific anti-O polysaccharide antibody response in Shiga toxin-producing *Escherichia coli*-associated hemolytic uremic syndrome (STEC-HUS) patients and its relevance for early diagnosis have not been previously studied in detail. In this pre-planned *post hoc* analysis, we evaluated the IgM/IgG antibody response in 55 patients monitored since the date of clinical STEC-HUS diagnosis. Four serum samples per patient were collected at 12–24 h (T1), 48 h after T1, and 7 and 28 days post-STEC-HUS diagnosis. The serogroup-specific anti-O polysaccharide antibody response was evaluated by the glycoprotein-based assays. *E. coli* O157, O145, O121, O103, O111, O26, and O45 Glyco-iELISAs and the lateral flow immunoassay (LFIA) CHEMSTRIP*E. coli* O157/O145 and three IgM/IgG response patterns were observed. Analysis of the serum samples at T1 by Glyco-iELISAs and the LFIA revealed an overall reactivity of 89.1% (49/55), a value significantly higher than those obtained by *stx*/Stx detection (74%, 37/50) and stool culture (16.3%, 9/55). When combining all methodologies, the association with STEC infection was 96.4% (53/55). In samples ranging from 2 to 5 days post-diarrhea onset, the reactivity by serology, *stx*/Stx detection, and stool culture was 77.8%, 73.1%, and 25.9%, respectively, but when combining all the methodologies, it increased to 92.6%. The detection of serogroup-specific anti-O polysaccharide antibodies alone or in combination with fecal diagnostics significantly contributed to STEC diagnosis in HUS patients, even very early in the course of the infection. These findings strongly support the incorporation of serology into diagnostic algorithms for timely diagnosis and proper management of STEC-HUS patients.

**IMPORTANCE:**

This study evaluated, for the first time, the dynamics of serogroup-specific IgM and IgG anti-O polysaccharide antibody response in 55 Shiga toxin-producing *Escherichia coli*-associated hemolytic uremic syndrome (STEC-HUS) pediatric patients. We provide evidence that the detection of serogroup-specific anti-O polysaccharide antibodies alone or in combination with bacterial isolation and/or stx/Stx detection from stool samples significantly contributes to STEC diagnosis in HUS patients in the first days after the onset of diarrhea. This could potentially improve the way we currently diagnose these infections. Our glycoprotein-based serological tests could be incorporated into diagnostic algorithms and implemented in primary care settings upon presentation of bloody or non-bloody diarrhea, enabling rapid and simple screening without the need for invasive procedures. This could allow the timely identification of these potentially harmful infections and close monitoring of these patients or help refer them to tertiary-care hospitals for proper clinical management.

**CLINICAL TRIALS:**

This study was registered with ClinicalTrials.gov as NCT05569746.

## INTRODUCTION

Shiga toxin (Stx)-producing *Escherichia coli* (STEC) are strains that produce Stx1 and/or Stx2 (1). These food- and water-borne pathogens have the potential to cause acute diarrhea (D) and bloody diarrhea (BD) and can trigger a thrombotic microangiopathy (TMA) that leads to a hemolytic uremic syndrome (HUS), a life-threatening disease characterized by the triad of hemolysis, platelet consumption, and acute kidney injury (AKI) (2).

The prevalence of STEC serogroups varies by region, with *E. coli* O157 being the most frequently identified in sporadic cases and outbreaks worldwide. However, in the last decades, HUS cases associated with non-O157 serogroups have increased (3). In the United States during 2010–2017, 71% of the reported outbreaks were caused by O157, and the most common non-O157 serogroups were O26 (36%), O111 (20%), and O121 (13%) (4). In countries of the European Union, O26 is the most frequently identified serogroup after O157 (5). In Argentina, the prevalence of the *E. coli* O157 serogroup ranges from 60% to 83%, and among non-O157, the most frequently detected serogroup is O145 (16.8%–28.75%), followed by O121 (3%–5.4%) and other serogroups (~10%) (6–10).

STEC-associated HUS (STEC-HUS) mainly affects children under 5 years old, being one of the leading causes of AKI in this pediatric population. This disease has a great social and economic impact because of its severity and long-term sequelae. BD occurs in about 90% of patients around 3–8 days after ingestion of contaminated food. After a median of 7 days, around 15%–20% of patients develop HUS, a proportion that can reach 30% during outbreaks (1, 11). Around 80% of patients with HUS require blood transfusion, and about 60% require dialysis. Apart from kidney injury, extrarenal complications (intestinal, cardiac, and neurological) may occur during the acute phase, with a mortality rate of 1.5% to 3%. Full recovery takes place in around 70% of the patients, but renal and neurological sequelae remain in 30% and 5% of the patients, respectively (12). In Argentina, STEC-HUS is endemic and exhibits the highest incidence globally, with a mortality rate of 2% to 4% (9). It is the main cause of AKI in children, an important cause of end-stage renal disease, and accounts for approximately 10% of kidney transplants in children and adolescents (13).

Today, there is no established specific therapy for STEC-HUS. INM004 is a medical product composed of the F(ab´)_2_ fragments from equine polyclonal antibodies that efficiently neutralize Stx1 and Stx2 by blocking the entrance of Stx to their target cells (14, 15). Phase 1 and phase 2 clinical trials showed that INM004 was well tolerated and was not associated with serious or severe adverse events (16). Furthermore, the efficacy results suggested a beneficial effect in the amelioration of kidney injury during the acute phase of the disease (17). These results supported the conduction of a phase 3 clinical trial which is in progress in different health centers in Argentina and Europe (18).

Currently, supportive nonspecific treatment is the only available option for the management of patients with STEC-HUS. INM004 therapy as well as monoclonal antibodies (19–21) that target Stx have shown promising preclinical and early clinical results. If STEC infection could be established in early stages of the disease, this would create a therapeutic window for both supportive care and/or specific antibody treatments. This is important because both treatments would be more efficient when administered earlier in the course of the disease (22–25). In addition, timely confirmation of the STEC infection is extremely valuable for STEC-HUS differential diagnosis from other forms of HUS, such as atypical HUS (26). The diagnosis of STEC infections can be made by fecal diagnostics and/or serological tests. Fecal diagnostics are performed from stool samples by the following: (i) bacterial isolation and characterization, (ii) detection of Free Fecal Stx (FFStx) by enzyme immunoassays, and/or (iii) detection of *stx* genes by polymerase chain reaction (PCR) (12, 27–29). Stool samples should be collected as soon as possible after the onset of diarrhea, as the bacterial load and Stx release decrease shortly after this. Instead, serum antibodies persist for several weeks and therefore may add value in the diagnosis of STEC (25). Serological tests include indirect Enzyme-Linked ImmunoSorbent Assays (ELISAs) that detect antibodies against the complete lipopolysaccharide (LPS) and glycoprotein-based ELISAs (Glyco-iELISAs) that specifically and exclusively detect antibodies against the O polysaccharide section of the LPS (30–32). Many authors reported that the detection of anti-LPS antibodies in combination with bacteriological methods and *stx*/Stx detection increases the evidence of STEC infection in HUS cases (33–36). However, false-positive reactions may occur because of cross-reactive antibodies against epitopes present in the core and lipid A moieties of LPS and shared by different STEC strains and other enterobacteria (37, 38). Instead, the glycoprotein-based assays use serogroup-specific antigens in which only the O polysaccharide section of the LPS is covalently linked to the carrier protein AcrA. These bacterial engineered glycoconjugates were developed for O157 and the “Big Six” STEC serogroups O26, O103, O111, O121, O145, and O45 by expressing the *Campylobacter jejuni* N-glycosylation machinery in *E. coli* (30, 32, 39). As previously demonstrated, these molecules show enhanced sensitivity and specificity for the serological detection of STEC O157, O145, and O121 infections in patients with a clinical diagnosis of HUS, offering potential improvements over conventional diagnostic methods (32). Subsequently, Wijnsma et al. demonstrated the benefits of using O157-glycoprotein compared to O157-LPS as antigens in an indirect ELISA due to its higher specificity and better assay performance (38). Moreover, the recent development of a glycoprotein-based *E. coli* O157/O145 LFIA allowed the detection of specific IgM antibodies very early during STEC infection, making it an ideal diagnostic tool to be implemented in pediatric emergency medical centers (31).

In this report, we aimed to evaluate the specific anti-O polysaccharide IgM/IgG antibody response and its relevance for early diagnosis in children with STECHUS enrolled in a phase 2 multicenter clinical trial conducted in Argentina to assess the safety, efficacy, and pharmacokinetics of INM004 (17). In a pre-planned *post hoc* analysis, we evaluated the antibody kinetics in 55 patients who were followed up for 28 days since the date of STEC-HUS diagnosis, and analyzed the contribution of the anti-O polysaccharide serological tests, either alone or in combination with fecal diagnostics, for early diagnosis of STEC infections.

## MATERIALS AND METHODS

### Study design and participants

Fifty-five patients aged between 1 and 8 years old with a clinical condition compatible with STECHUS were enrolled from 10 October 2022 to 30 April 2023 as part of the phase 2 multicenter study CT-INM004-03 conducted to assess the safety, efficacy, and pharmacokinetics of INM004 in pediatric patients with STEC-HUS (17). This study was prospectively registered on clinicaltrials.gov (NCT05569746).

This study was conducted in 13 participating Health Centers in different regions of Argentina. As an inclusion criterion, patients must have been hospitalized and have a history of diarrhea onset within 13 days prior to diagnosis of STEC-HUS at the participating institution. The Centers were in Buenos Aires City (4), Buenos Aires Province (4), Córdoba (2), Mendoza (1), Santa Fe (1), and La Pampa (1).

STEC-HUS defining diagnosis included kidney damage, and either hemolysis or decreased platelets. Kidney damage was defined as serum creatinine above the upper limit of normal (ULN) for age and sex, and/or hematuria (≥5 red blood cells per field or ≥27 red blood cells/μL in urinary sediment); hemolysis, as lactate dehydrogenase above ULN for age and sex and/or schistocytes in peripheral blood spread; and platelet consumption, as platelet count <150 × 10^3^/μL in peripheral blood and/or ≥50% decrease in peripheral blood platelet count from baseline or within the previous 24 h.

The patients received two doses of INM004, with an interval of 24 h, and were followed up for 28 days since the date of STEC-HUS diagnosis, which was defined as Day 0 (T0) for the assessment of clinical manifestations and laboratory parameters.

A study-specific electronic Case Report Form (eCRF) was used as the only tool for collection, storage, and reporting of data. Recorded data consisted of demographic and baseline disease characteristics, including prodromal history, clinical history associated with STEC-HUS (renal and extra-renal involvement), general medical history, STEC diagnosis, concurrent conditions at diagnosis, dialysis requirement, and length of hospitalization (17). Laboratory findings, vital signs, adverse events, and concomitant medication, among other variables, during the 28-day follow-up were also recorded.

### STEC diagnosis

**Fig 1:**
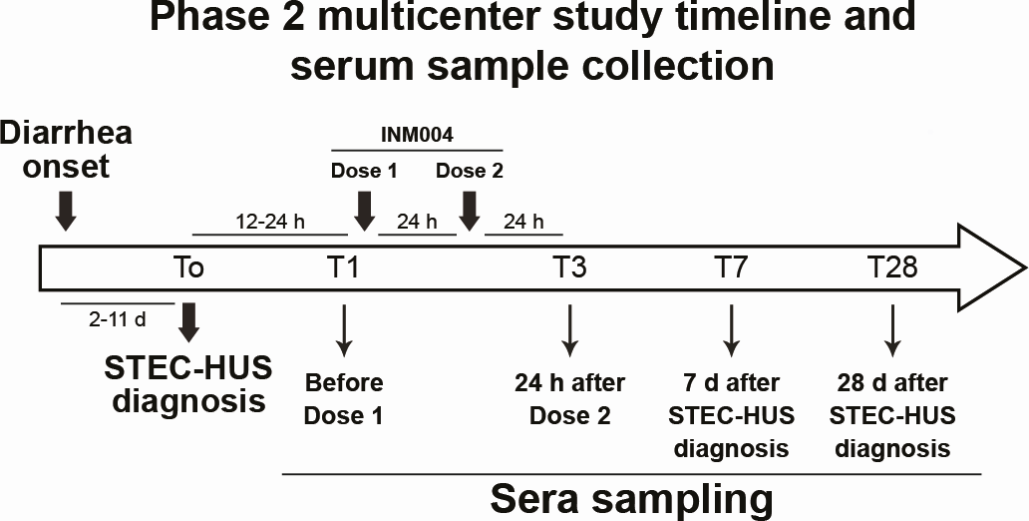
Experimental layout of the INM004 phase 2 clinical trial and the time points of serum sampling. T0 corresponds to the date of STEC-HUS diagnosis according to the criteria described in Material and Methods. Dose 2 of INM004 was administered 24 h after dose 1. Four serum samples were collected for each patient: T1; 12 to 24 h after STEC-HUS diagnosis and before dose 1 administration of INM004; T3, 48 h after T1 and 24 h after dose 2 administration of INM004; T7, 7 days after STEC-HUS diagnosis; and T28, 28 days after STEC-HUS diagnosis.

Fecal samples were collected upon hospital admission as part of the standard of care (SoC). Dates of specimen collection and processing of the stool sample in the local laboratory, type of sample (fresh stool or rectal swab), and result of the microbiological tests were recorded. STEC diagnosis was performed following the SoC of each center, applying the available method/s at the site (Table S1). These methodologies included detection of the following: (i) generic Stx, Stx1, and/or Stx2 by enzyme immunoassay (1/13 centers); (ii) *stx* genes by PCR (conventional PCR, RT-PCR, or FilmArray; 12/13 centers); (iii) free fecal Shiga toxin on Vero cells (FFStx, 3/13 centers), and/or (iv) stool culture and characterization (8/13 centers). Four serum samples were collected for each patient at the following time points: 12 to 24 h after STEC-HUS diagnosis and before dose 1 administration of INM004 (T1), 48 h after T1 (T3) (dose 2 of INM004 was administrated 24 h after dose 1), 7 days after STEC-HUS diagnosis (T7), and 28 days after STEC-HUS diagnosis (T28) (Fig. 1). The serum samples were collected and stored in each center and then sent to Chemtest Argentina S. A. for the serological analysis. The serological tests were performed using the glycoprotein-based enzyme-linked immunosorbent assays CHEMLIS *E. coli* O157, O145, O121, and O103 Glyco-iELISAs (Chemtest Argentina S. A.) and the glycoprotein-based immunochromatographic test CHEMSTRIP *E. coli* O157/O145 (Chemtest Argentina S. A.), according to the manufacturers’ instructions. CHEMLIS and CHEMSTRIP products were registered by Chemtest Argentina S. A. as IVDs in Argentina at Administración Nacional de Medicamentos, Alimentos y Tecnología Médica (ANMAT), the national health authority for the registration and authorization of these types of products. The in-house Glyco-iELISAs for *E. coli* O26, O111, and O45 serogroups were performed as previously described (30). The Glyco-iELISAs allow the detection of specific IgM and IgG antibodies directed exclusively against the corresponding O polysaccharide (O157, O145, O121, O103, O26, O111, or O45 polysaccharide) (30, 32, 38), and the results were expressed as the percentage of reactivity (PR) with respect to the positive control included in each assay. The LFIA CHEMSTRIP *E. coli* O157/O145 allows the rapid detection (10 min) of IgM antibodies against the O-polysaccharide of the LPS of *E. coli* O157 and O145 in serum, plasma, or whole blood obtained by venipuncture or finger/heel capillary puncture (31). LFIA results were expressed as the color intensity (CI) of the TL determined by visual inspection and registered based on an ascending color intensity scale from 0 to 10, where CI = 0 indicates a negative result and CI > 0 a positive result. LFIA readers were blinded, and the analysis was carried out by two independent people.

## RESULTS

### Serological follow-up by Glyco-iELISA

**Fig 2:**
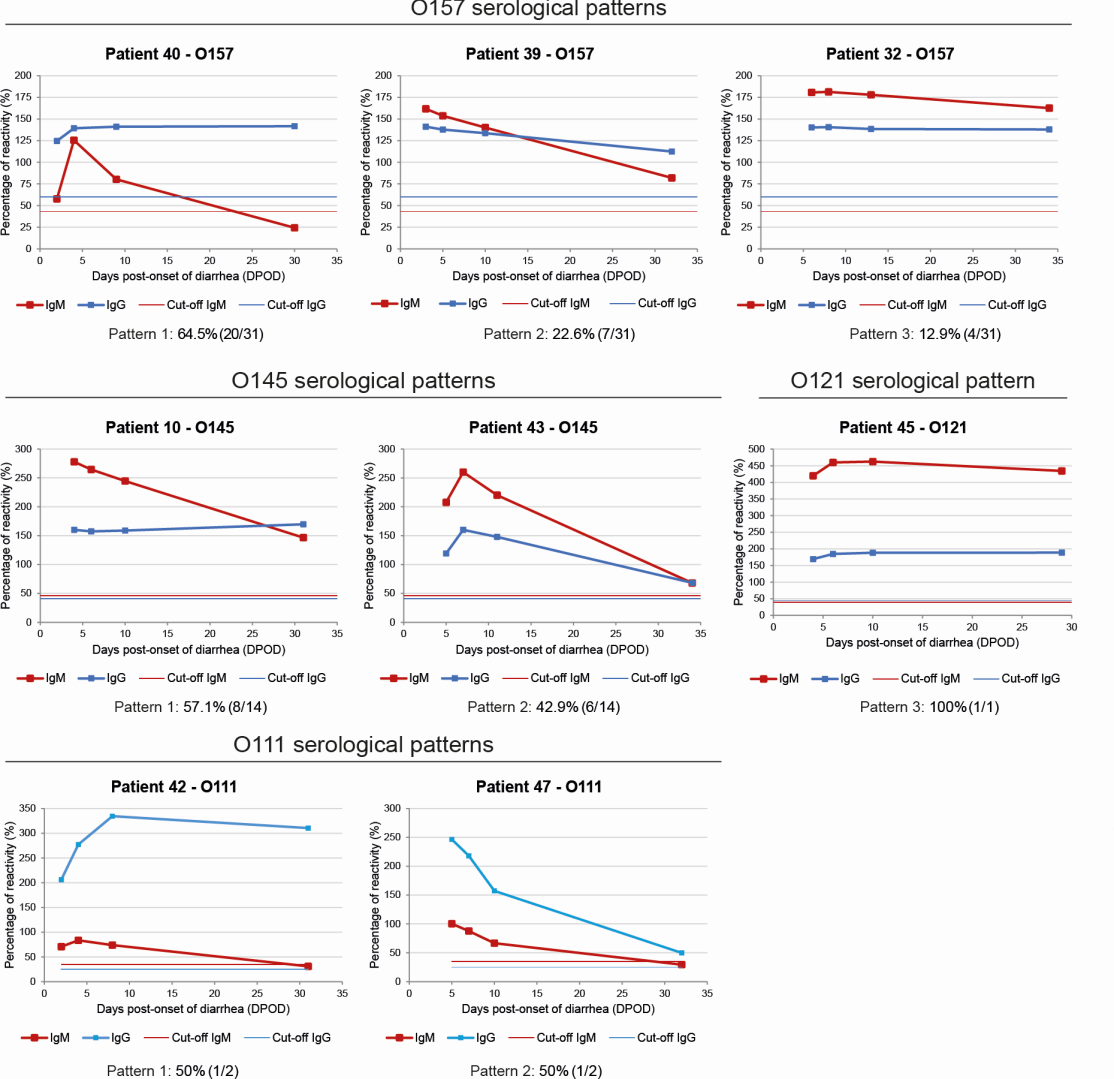
Serological follow-up by Glyco-iELISAs. The serum samples obtained at T1, T3, T7, and T28 were analyzed by the CHEMLIS *E. coli* O157, O145, O121, O103, O26, O111, and O45 Glyco-iELISAs. The percentage of reactivity for specific IgM (red curves) and IgG (blue curves) antibodies was graphed as a function of the days post-onset of diarrhea (DPOD), calculated as the days elapsed between the onset of diarrhea and the date of sample collection. The horizontal red and blue lines mark the cutoff values for IgM and IgG, respectively. The results obtained for the eight patients, representing the different observed serological patterns for O157 and O145, the two positive patients for O121, and the only positive patient for O121, are shown (serological response curves for all patients are shown in Fig. S1). The percentage of patients with the corresponding serological pattern is indicated below the graph.

To evaluate the antibody response in pediatric patients with a diagnosis compatible with STECHUS, 4 consecutive serum samples obtained from 55 patients enrolled as part of the phase 2 clinical trial CT-INM004-03 were analyzed (Fig. 1). The patients were followed up for 28 days since the date of STEC-HUS diagnosis (T0), and sera were obtained at the following time points: 12 to 24 h after STEC-HUS diagnosis (T1); 48 h after T1 (T3); and 7 and 28 days after STEC-HUS diagnosis (T7 and T28). The serum samples obtained at T1, T3, T7, and T28 were analyzed by the *E. coli* O157, O145, O121, O103, O26, O111, and O45 Glyco-iELISAs, and the reactivity values for specific IgM and IgG antibodies were plotted as a function of the days post-onset of diarrhea (DPOD) calculated as the days elapsed between the onset of diarrhea and the date of sample collection (Fig. S1; Table S2). Based on this analysis, three serological patterns were identified: pattern 1, IgM reactivity decreased over time, but IgG reactivity was maintained; pattern 2, IgM and IgG reactivities decreased over time; and pattern 3, IgM and IgG reactivities were maintained. Figure 2 shows the antibody curves representative of each of the patterns identified for each serogroup. Most of the O157-positive patients showed pattern 1 (64.5%, 20/31) followed by patterns 2 (22.6%, 7/31) and 3 (12.9%, 4/31). For O145-positive patients, serological patterns 1 (57.1%, 8/14) and 2 (42.9%, 6/14) were observed. The two O111-positive patients followed patterns 1 and 2, and the O121-positive patient showed pattern 3. No statistically significant differences were found between the evaluated clinical variables (baseline glomerular filtration rate, number of days of diarrhea until STEC-HUS diagnosis, days on dialysis, and length of hospitalization) and the three identified serological pattern groups, as analyzed by Kruskal-Wallis tests (data not shown).

**Fig 3:**
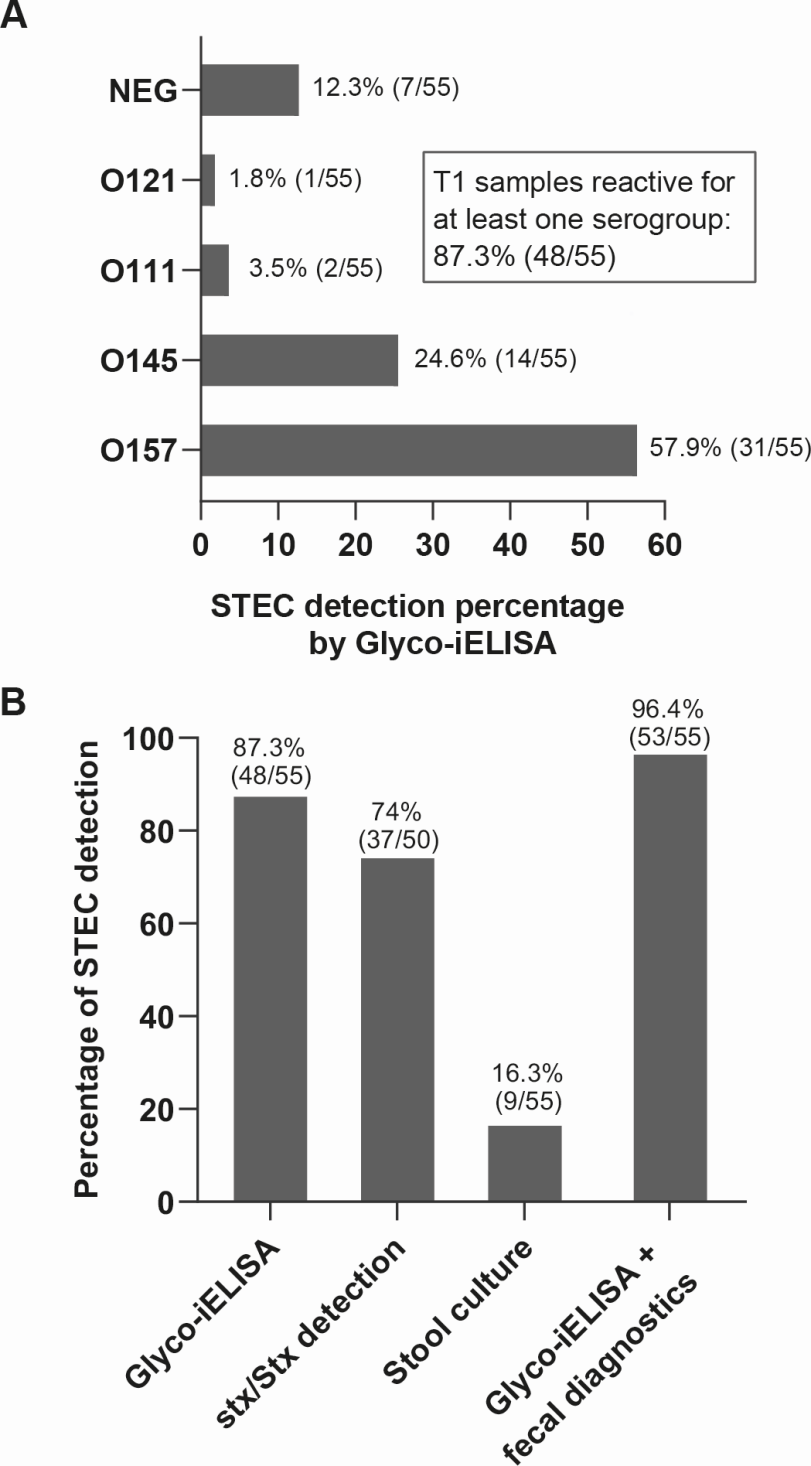
Analysis of T1 serum samples and comparison with fecal diagnostics. (**A**) Analysis of the samples at T1 by Glyco-iELISAs. The serum samples of the 55 patients obtained at T1 were analyzed by Glyco-iELISAs for the detection of IgM and IgG serogroup-specific antibodies against the O157, O145, O121, O103, O111, O26, and O45 polysaccharides, and the detection percentage was calculated with respect to the total number of samples (*n* = 55). The overall reactivity by serology reached a percentage of 87.3% (48/55). POS, positive; NEG, negative. (**B**) Comparative analysis of STEC detection according to the diagnostic methodology used. The detection percentage was calculated based on the analysis of the stool samples collected upon admission and the T1 serum samples. Glyco-iELISA, IgM detection by Glyco-iELISAs; *stx*/Stx detection, FFStx Shiga toxin detection and/or *stx* gene detection by PCR (Shiga toxin detection was not performed in the samples from five patients); stool culture, bacterial isolation; and Glyco-iELISA + fecal diagnostics, STEC detection combining all the above methodologies.

Analysis of the Glyco-iELISAs results at T1 revealed an overall IgM reactivity of 87.3% (Fig. 3A), indicating that in 48 of the 55 patients, HUS could be associated with a STEC acute infection. In these 48 positive patients, the prevalence of STEC serogroups was similar to that previously reported in Argentina (6), with O157 being the predominant serogroup (31/55, 57.9%), followed by O145 (14/55, 24.6%), O111 (2/55, 3.5%), and O121 (1/55, 1.8%) (Fig. 3A). Only the T1 sample of patient 51 was positive for IgM but negative for IgG; nevertheless, T3, T7, and T28 samples of this patient became IgG-positive (Fig. S1; Table S2). For 7 of the 55 HUS patients (patients 17, 21, 22, 29, 37, 50, and 53), both the T1 sample and the subsequent ones (T3, T7, and T28) were serologically negative by Glyco-iELISAs (Fig. 3A; Table S2). All these samples were also negative by bacterial isolation from stool samples. However, the detection of *stx*/Stx was positive for 4 of these patients (22, 37, 50, and 53) and negative for the other three patients (17, 21, and 29). The T1 sample from Patient 29 yielded indeterminate IgM and IgG results by O145 Glyco-iELISA, but tested positive by O145 LFIA (see below). Based on these results, it is most likely that in the 6 patients for which serology was negative (17,21, 22, 37, 50, and 53), HUS has been caused by a serogroup of STEC different from those tested by ELISA and LFIA. According to the epidemiological surveillance reports from the Ministry of Health in Argentina, serogroups O157, O145, O121, and O103 account for 91% of the 151 STEC infections studied at the National Reference Laboratory in 2024, including HUS and diarrhea cases. Other serogroups reported were O111 (1%), O104 (1%), O48 (1%), O85 (1%), O151/O118 (1%), and O91 (2%) (40). However, we cannot rule out the possibility that they are true false negatives of the tests, although, as mentioned above, they were not only negative for the first sample (T1) but also for the samples taken at subsequent time points (T3, T7, and T28). Finally, none of the six patients had features suggestive of non-STEC HUS.

In order to compare the sensitivity of the serological tests with fecal diagnostics, the percentage of STEC identification by *stx*/Stx detection (FFStx and/or PCR) and by bacterial isolation from stool samples was also calculated, reaching the reactivity values of 74% (37/50) and 16.3% (9/55), respectively (Fig. 3B). These values were significantly lower than the reactivity observed by Glyco-iELISA at T1 (87.3%, 48/55). Furthermore, combining serological tests with fecal diagnostics, HUS could be associated with STEC infection in 53 of the 55 patients (96.4%) (Fig. 3B).

Taken together, these results demonstrate that the detection of serogroup-specific anti-O polysaccharide antibodies, alone or in combination with fecal diagnostics, significantly contributes to STEC diagnosis in HUS patients.

### Serological analysis by LFIA

**Fig 4:**
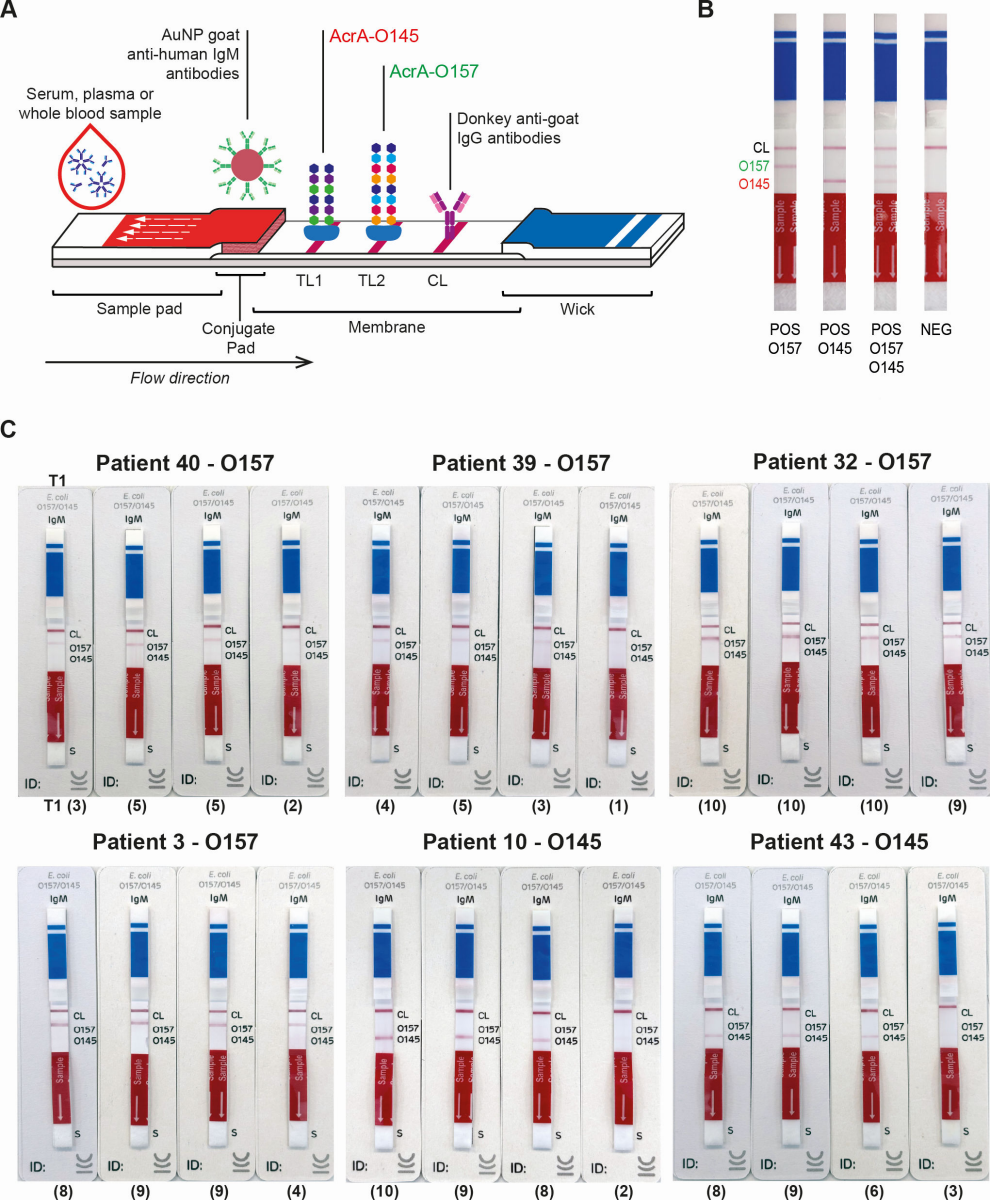
Serological follow-up by the immunochromatographic test CHEMSTRIP *E. coli* O157/O145. (**A**) Principle of the assay. The serum sample is placed on the sample pad, and after adding the running buffer, the liquid phase will flow through the strip to the absorbent pad (black arrow) and resuspend the colloidal gold nanoparticles (AuNPs) labeled with goat anti-human IgM antibodies (AuNP-conjugate). The conjugate binds to all IgM antibodies present in the sample, forming a complex that migrates through the membrane. If the sample contains anti-O157 and/or anti-O145 polysaccharide IgM antibodies, the nanoparticle-antibody complexes are captured by the AcrA-O157 and/or AcrA-O145 antigens immobilized at the TL1 and TL2, respectively, and visualized as a red/purple line. Independently of the presence of anti-O157 and/or anti-O145 antibodies, the AuNP-conjugate in excess continues running through the strip and is captured by the anti-goat IgG antibodies immobilized on the CL. The result is read after 10 min by visual inspection for staining of the test and control lines. (**B**) Interpretation of the results. The absence of the CL or any result obtained after the indicated reading time invalidates the assay. (**C**) Pictures of the test strips showing the results of the serological follow-up for 6 patients representative of the 55 analyzed. The serum samples obtained at T1, T3, T7, and T28 were analyzed by the CHEMSTRIP *E. coli* O157/O145 immunochromatographic test. Results are expressed as the color intensity (CI) of the TL determined by visual inspection and registered based on an ascending color intensity scale from 0 to 10. Interpretation of the result: CI = 0, negative (NEG); CI > 0, positive (POS). The numbers below the strips indicate the CI of the corresponding TL for each strip.

Recently, we have developed and validated a multiplex LFIA for the rapid (10 min) detection of specific IgM antibodies against the O polysaccharide of *E. coli* O157 and O145 serogroups, the most prevalent *E. coli* serogroups associated with BD and HUS in Argentina (31). The CHEMSTRIP*E. coli* O157/O145 LFIA combines the power of immunochromatographic technology for point-of-care diagnosis with bacterial glycoengineering technology to produce bacterial-engineered glycoconjugates (recombinant glycoproteins AcrA-O157 and AcrA-O145) used as antigens. The principle of the assay and how the results should be interpreted are shown in Fig. 4A and B.

Analysis of the serum samples with CHEMSTRIP *E. coli* O157/O145 revealed a high agreement with the results obtained by Glyco-iELISAs (Table S2). Pictures of the test strips showing the results of the serological follow-up for 6 patients who are representative of the 55 analyzed are shown in Fig. 4C. All the T1 samples positive for O157 and O145 by Glyco-iELISA were also positive by the LFIA (Table 1). As expected, all the negative samples, except one, and the positive samples for O111 and O121 by Glyco-iELISAs were negative by the LFIA. The exception was the T1 sample from patient 29, which was indeterminate by O145 Glyco-iELISA but positive for O145 by the LFIA (Table 1; Table S2). Therefore, the STEC detection rate by LFIA was 83.6% (46/55).

**TABLE 1:** Analysis of T1 samples by CHEMSTRIP *E. coli* O157/O145 and comparison with Glyco-iELISA results

Glyco-iELISA	CHEMSTRIP *E. coli* O157/O145		
	O157-positive	O145-positive	Negative
O157-positive (*n* = 31)	31	0	0
O145-positive (*n* = 14)	0	14	0
O121-positive (*n* = 1)	0	0	1
O111-positive (*n* = 2)	0	0	2
Negative (*n* = 7)	0	1^*a*^	6
Total (*n* = 55)	31	15	9

^*a*^ Sample corresponding to patient 29 with an indeterminate result for IgM O145 by Glyco-iELISA.

### STEC detection according to the days after the onset of diarrhea

To evaluate the sensitivity of the anti-O polysaccharide serological tests for the diagnosis of STEC infection at early stages of infection, we analyzed the Glyco-iELISA and LFIA results according to the days elapsed between the onset of diarrhea and sample collection at T1 (days post-onset of diarrhea, DPOD) and compared them with fecal diagnostics. All the patients enrolled in this study had a history of diarrhea onset within 11 days prior to diagnosis of STEC-HUS, and for all of them, we had very reliable information about the days elapsed between the onset of diarrhea, with or without blood, and the collection of the first serum sample (T1) (Fig. 1). Complete information and results of T1 samples sorted according to the DPOD are shown in Table S3.

**Fig 5:**
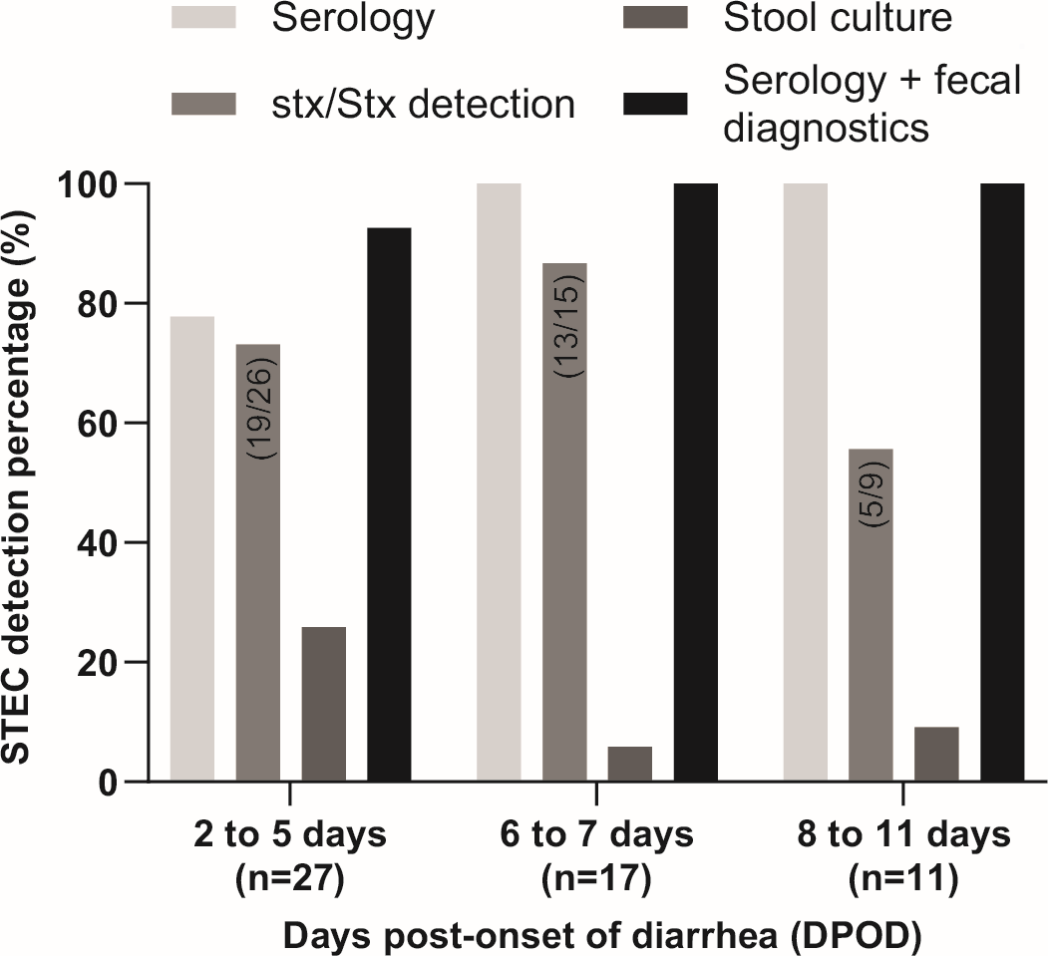
STEC detection according to the days elapsed between the onset of diarrhea and sample collection at T1 (days post-onset of diarrhea, DPOD), and comparative analysis with fecal diagnostics. Serology, IgM and/or IgG detection by Glyco-iELISAs and CHEMSTRIP *E. coli* O157/O145; *stx*/Stx detection, FFStx and/or *stx* gene detection by PCR; stool culture, bacterial isolation and characterization; and Glyco-iELISA + fecal diagnostics, STEC detection combining all the above methodologies. The number of samples analyzed for each interval time is indicated in parentheses. *stx*/Stx detection was not performed for the samples from 5 patients; one of the interval time 2 to 5 DPOD, two of 6 to 7 DPOD, and two of 8 to 11 DPOD.

As shown in Fig. 5, in the 27 T1 samples obtained between 2 and 5 DPOD, the percentage of detection by serology, including the Glyco-iELISAs and the LFIA, was 77.8% (21/27). This value was higher than those obtained by *stx*/Stx detection (73.1%, 19/26) and bacterial isolation from stool samples (25.9%, 7/27). Furthermore, combining serology with fecal diagnostics, the sensitivity reached a value of 92.6%, allowing the detection of STEC in 25 of the 27 patients at this time range. As mentioned above, in the six patients serologically negative for this time interval, the result is unrelated to how early the T1 serum sample was obtained since for the samples collected subsequently (T3, T7, and T28), the serology also resulted negative. Interestingly, from 6 DPOD onward, the percentage of detection by serology increased to 100%. By *stx*/Stx detection and bacterial isolation, the percentages of reactivity for the time interval 6 to 7 days were 76.5% and 5.9%, and for the time range of 8 to 11 days, these values were 45.5% and 9.1%, respectively.

These results demonstrate that the detection of serogroup-specific anti-O polysaccharide antibodies alone or in combination with fecal diagnostics significantly increases the detection of STEC in HUS patients during the early stages of the infection.

## DISCUSSION

STEC-HUS is a serious and life-threatening disease in young children. Due to the characteristics of the infection and the symptoms, as well as the rapid progression of the disease, early etiological diagnosis is central for patient management and differential diagnosis between typical and atypical HUS. Timely and accurate diagnosis allows physicians to take critical decisions such as promoting hospital admissions and close monitoring of the patient with a more thorough battery of clinical and laboratory evaluations, avoiding delays in initiating a supportive treatment—particularly in the early stages of the disease—taking contact precautions to minimize secondary spread within the community, and avoiding the use of antibiotic therapy that can increase the risk of evolving into HUS (1, 41).

The widespread use of molecular methods in recent years has facilitated the early detection of *stx* genes, and PCR-based STEC diagnostics have been proposed to improve the management of BD patients and to monitor the potential progression to HUS (42). However, diagnosis from stool samples is challenging because bacterial load declines quickly after the first symptoms and there is limited availability of fast, easy-to-deploy, and accurate diagnostic tests. This second point is crucial, as the current diagnostic assays to identify STEC infections are either time-consuming, expensive, and difficult to implement in many laboratory settings or have low sensitivity. We have shown that the serological diagnosis of the disease using recombinant glycoproteins significantly increases the efficacy of the diagnosis (31, 32) and, in Argentina, the Glyco-ELISAs have been incorporated since 2016 into the diagnostic algorithm of the National Reference Laboratory, with excellent results (6, 10). Even though the performance of these Glyco-ELISAs, and more recently LFIA tests, has been well documented over the past years (8, 9, 31, 38), the kinetics of antibodies has not been studied, and we still lack information to narrow down how early after the onset of the diarrhea these assays are still sensitive. This last issue is particularly important as it has been demonstrated that the earlier the supportive care is established during the infection, the better the short- and long-term prognosis of patients with STEC-associated HUS (22–24, 43).

In this report, we evaluated for the first time the serogroup-specific IgM and IgG anti-O polysaccharide antibody response over time, according to the days post-onset of diarrhea (bloody or not), in 55 pediatric patients with a diagnosis compatible with STECHUS. Three serological patterns were identified, and in all the positive patients, the first serum sample collected (T1) was IgM-positive, demonstrating the value of IgM determination for the diagnosis of acute STEC infections. In this sense, the Glyco-iELISAs and LFIA results at T1 revealed an overall IgM reactivity of 89.1% (49/55), a value significantly higher than those observed by *stx*/Stx detection (74%, 37/50) and bacterial isolation (16.3%, 9/55) from stool samples. It must be noted that fecal tests’ performance was not available for all patients given the diversity in SoC among the centers; also, the negative results obtained may have been affected by the timeline for sample collection (8/18, 44.4%, were collected after 7 days from diarrhea onset), antibiotic treatment (5/18, 27.8%), and the use of rectal swab instead of fresh stool (4/18, 22.2%). Since these situations may still occur due to different reasons, serology tests become especially valuable. In this sense, when serology was combined with fecal diagnostics, the sensitivity reached a value of 96.4% (53/55), demonstrating the contribution of the glycoprotein-based serological tests to STEC diagnosis in HUS patients. Even though the LFIA assay employed in this study enables the detection of IgM antibodies against O157 and O145 O-polysaccharides, which represent the most prevalent STEC serogroups in Argentina, this assay could be readily adapted for the identification of additional clinically relevant serogroups, including O26, O103, O111, O121, O145, and O45 (30). Moreover, the flexibility of the bacterial glycoengineering platform used to produce these glycoprotein-based antigens offers a powerful strategy to generate novel antigens, thereby facilitating the development of region-specific diagnostic ELISA and LFIA tools targeting epidemiologically relevant serogroups worldwide.

Because of the importance of timely diagnosis of STEC-HUS, we evaluated the sensitivity of the serological tests based on the days after the onset of diarrhea. Our glyco-tests showed excellent sensitivity very shortly after the onset of diarrhea in HUS patients; between the 2 and 5 days post-onset of the diarrhea stage, the serological tests have almost 80% sensitivity and, if combined with other diagnostic methods, can reach 93%. After the 5th day, the sensitivity of our serological assays is 100%. In the six patients for which HUS could not be associated with STEC by serology, both the T1 sample and the subsequent ones (T3, T7, and T28) were negative by Glyco-iELISAs and LFIA, indicating that the negative results were not due to the sample being taken too early but most probably due to the fact that HUS was caused by other serogroups different from those analyzed. Furthermore, the IgM antibody response curves showed that for most of the patients, the reactivity values observed with the first serum sample (T1) were at or near the peak of the curve (see Fig. S1), strongly suggesting that STEC diagnosis could have also been confirmed with a sample taken earlier. To our knowledge, this is the first study monitoring the serological response with a set of patients in which the exact timeline of development of the disease is known. In all these cases, we can precisely track down the onset of the diarrhea and, thus, be confident that the time the sample was collected was precise. This is different from other studies that used retrospective samples of patients that developed HUS, as in almost all these cases, the exact timeline of the disease was not well known.

Our findings indicate that, upon clinical presentation, a presumptive diagnosis of STEC infection can be made very early, quickly, and easily using a simple, non-invasive blood drop. This could potentially change the way we currently identify these infections. We propose that our serological tests could be adopted in healthcare settings, allowing for easy screening of patients presenting with either bloody or non-bloody diarrhea, without the need for invasive procedures. This would enable the identification of these potentially harmful infections and facilitate closer monitoring for appropriate clinical management in case of HUS progression and timely referral if necessary.

## Data Availability

All data and materials necessary to reproduce the findings of this study are comprehensively documented within the main paper and its supplemental material.

## SUPPLEMENTAL MATERIAL

Fig. S1 jcm.01415-25-s0001.pdf Serological follow-up by Glyco-iELISAs.

Table S1 jcm.01415-25-s0002.pdf Diagnostic methodologies used in each of the centers that participated in the study.

Table S2 jcm.01415-25-s0003.pdf Complete information and results of the samples analyzed in this study.

Table S3 jcm.01415-25-s0004.pdf Complete information and results of T1 samples sorted according to the days post-onset of diarrhea (DPOD).

ASM does not own the copyrights to Supplemental Material that may be linked to, or accessed through, an article. The authors have granted ASM a non-exclusive, world-wide license to publish the Supplemental Material files. Please contact the corresponding author directly for reuse.
