# Supplementary material for: Serology as an early diagnostic tool in pediatric patients with Shiga toxin-producing Escherichia coli-associated hemolytic uremic syndrome: a post hoc analysis of a phase 2 clinical trial
Source: J Clin Microbiol. 2026 Feb 27;64(4):e01415-25. doi: 10.1128/jcm.01415-25 (PMC13059726; doi:10.1128/jcm.01415-25)
Supplement: Fig. S1 — Serological follow-up by Glyco-iELISAs. [file jcm.01415-25-s0001.pdf]

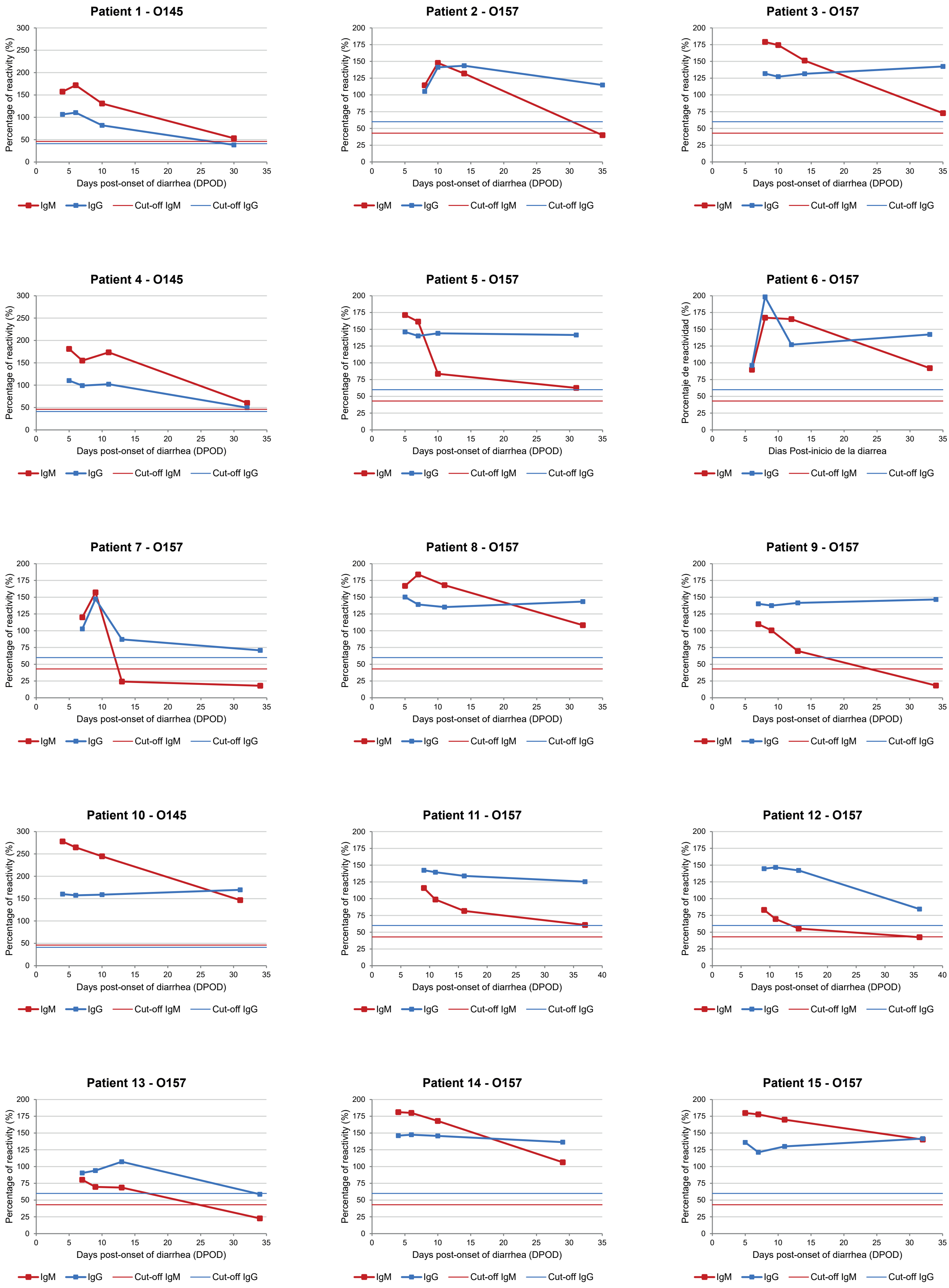
Patient 1 - O145
Patient 2 - O157
Patient 3 - O157
Patient 4 - O145
Patient 5 - O157
Patient 6 - O157
Patient 7 - O157
Patient 8 - O157
Patient 9 - O157
Patient 10 - O145
Patient 11 - O157
Patient 12 - O157
Patient 13 - O157
Patient 14 - O157
Patient 15 - O157
Percentage of reactivity (%)
Porcentaje de reactividad (%)
Days post-onset of diarrhea (DPOD)
Dias Post-inicio de la diarrea
IgM
IgG
Cut-off IgM
Cut-off IgG

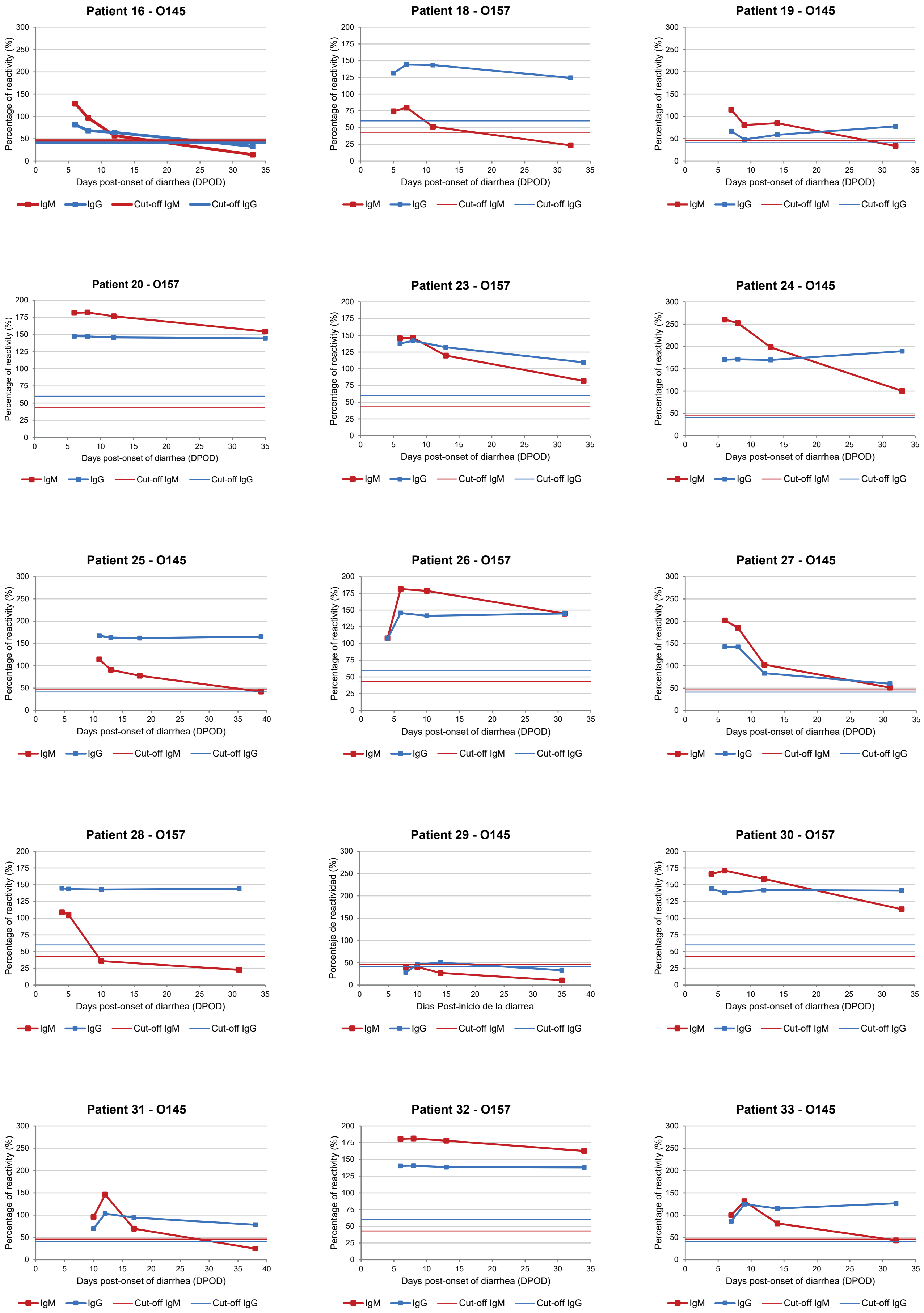
Patient 16 - O145
Patient 18 - O157
Patient 19 - O145
Patient 20 - O157
Patient 23 - O157
Patient 24 - O145
Patient 25 - O145
Patient 26 - O157
Patient 27 - O145
Patient 28 - O157
Patient 29 - O145
Patient 30 - O157
Patient 31 - O145
Patient 32 - O157
Patient 33 - O145
Percentage of reactivity (%)
Porcentaje de reactividad (%)
Days post-onset of diarrhea (DPOD)
Dias Post-inicio de la diarrea
IgM
IgG
Cut-off IgM
Cut-off IgG

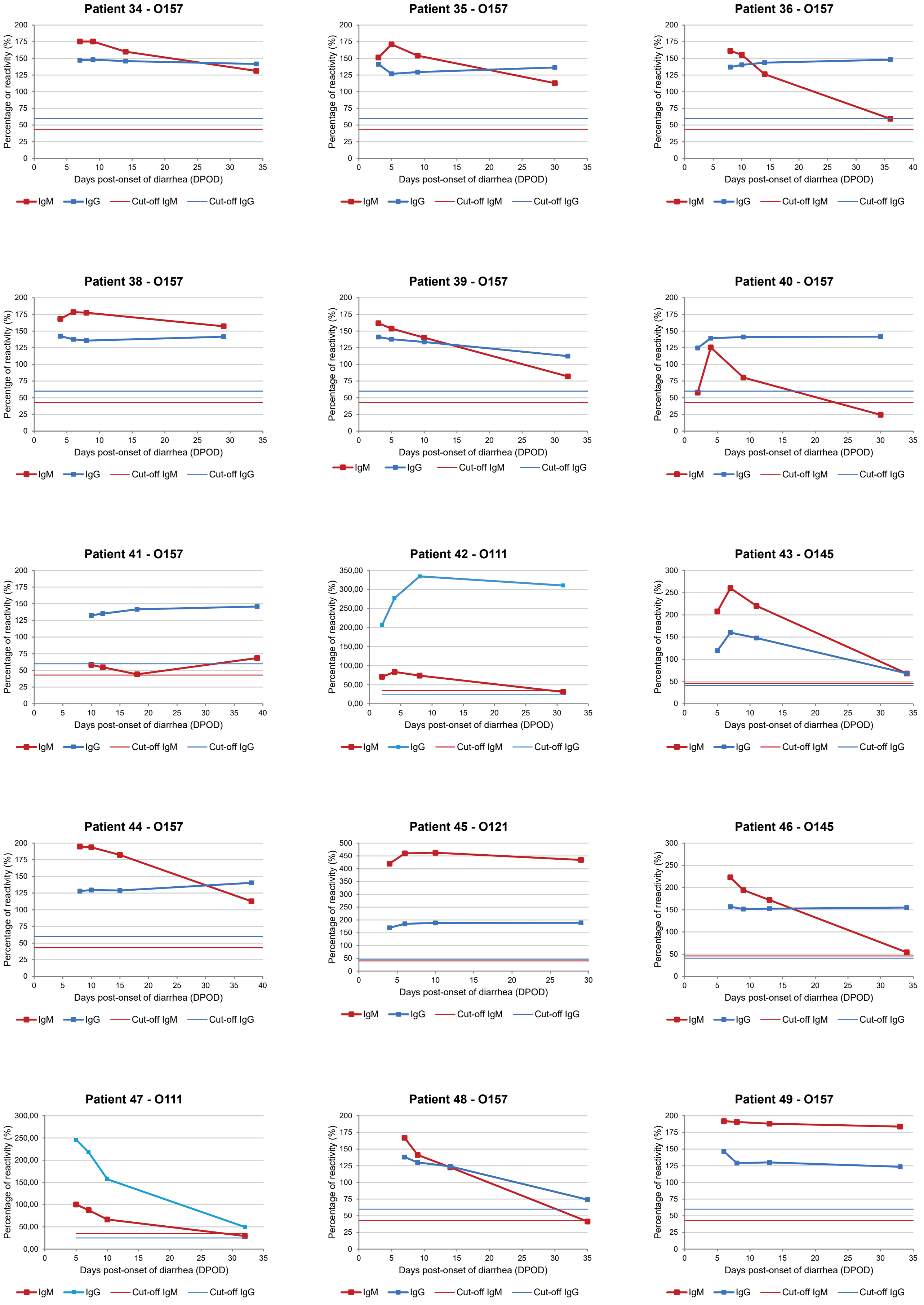
Patient 34 - O157
Percentage or reactivity (%)
Days post-onset of diarrhea (DPOD)
IgM
IgG
Cut-off IgM
Cut-off IgG
Patient 35 - O157
Percentage of reactivity (%)
Days post-onset of diarrhea (DPOD)
IgM
IgG
Cut-off IgM
Cut-off IgG
Patient 36 - O157
Percentage of reactivity (%)
Days post-onset of diarrhea (DPOD)
IgM
IgG
Cut-off IgM
Cut-off IgG
Patient 38 - O157
Percentage of reactivity (%)
Days post-onset of diarrhea (DPOD)
IgM
IgG
Cut-off IgM
Cut-off IgG
Patient 39 - O157
Percentage of reactivity (%)
Days post-onset of diarrhea (DPOD)
IgM
IgG
Cut-off IgM
Cut-off IgG
Patient 40 - O157
Percentage of reactivity (%)
Days post-onset of diarrhea (DPOD)
IgM
IgG
Cut-off IgM
Cut-off IgG
Patient 41 - O157
Percentage of reactivity (%)
Days post-onset of diarrhea (DPOD)
IgM
IgG
Cut-off IgM
Cut-off IgG
Patient 42 - O111
Percentage of reactivity (%)
Days post-onset of diarrhea (DPOD)
IgM
IgG
Cut-off IgM
Cut-off IgG
Patient 43 - O145
Percentage of reactivity (%)
Days post-onset of diarrhea (DPOD)
IgM
IgG
Cut-off IgM
Cut-off IgG
Patient 44 - O157
Percentage of reactivity (%)
Days post-onset of diarrhea (DPOD)
IgM
IgG
Cut-off IgM
Cut-off IgG
Patient 45 - O121
Percentage of reactivity (%)
Days post-onset of diarrhea (DPOD)
IgM
IgG
Cut-off IgM
Cut-off IgG
Patient 46 - O145
Percentage of rreactivity (%)
Days post-onset of diarrhea (DPOD)
IgM
IgG
Cut-off IgM
Cut-off IgG
Patient 47 - O111
Percentage of reactivity (%)
Days post-onset of diarrhea (DPOD)
IgM
IgG
Cut-off IgM
Cut-off IgG
Patient 48 - O157
Percentage of reactivity (%)
Days post-onset of diarrhea (DPOD)
IgM
IgG
Cut-off IgM
Cut-off IgG
Patient 49 - O157
Percentage of reactivity (%)
Days post-onset of diarrhea (DPOD)
IgM
IgG
Cut-off IgM
Cut-off IgG

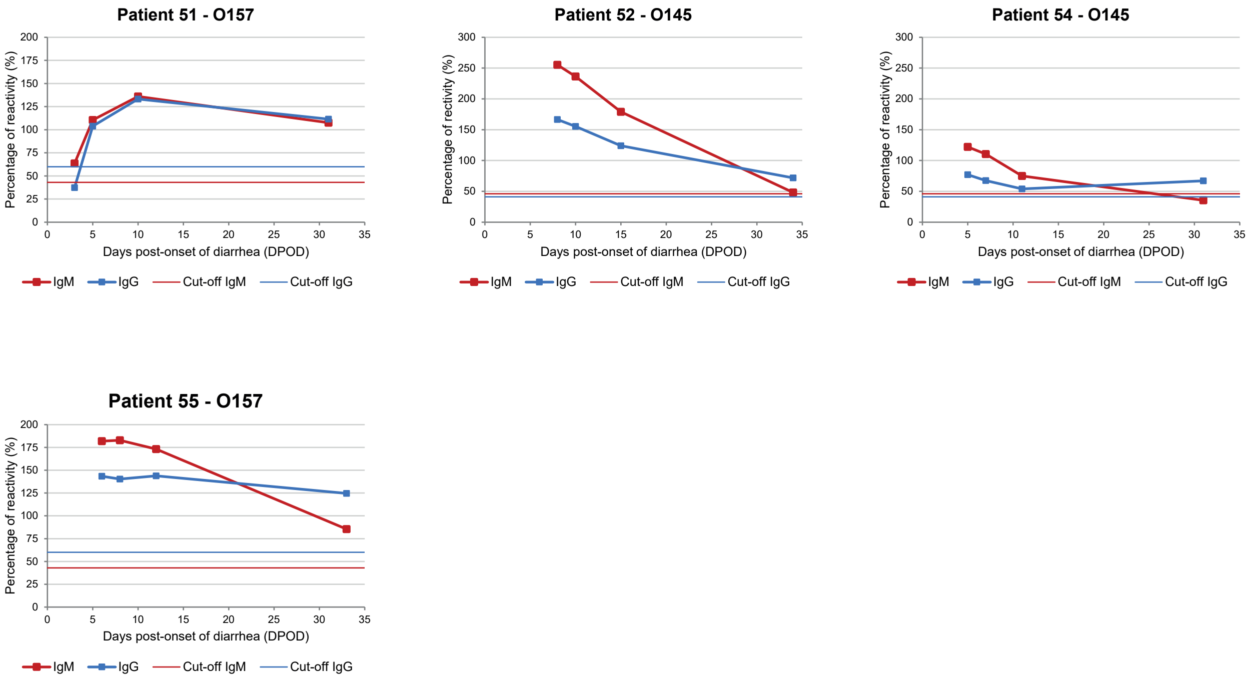

Figure S1. Serological follow-up by Glyco-iELISAs. The serum samples obtained at T1, T3, T7 and T28 for each patient were analyzed by the CHEMLIS® E. coli O157, O145, O121, O103, O26, O111 and O45 Glyco-iELISAs. The percentage of reactivity for specific IgM (red curves) and IgG (blue curves) antibodies were graphed as a function of the days post onset of diarrhea (DPOD) calculated as the days elapsed between the onset of diarrhea and the date of sample collection. The horizontal red and blue lines mark the cutoff values for IgM and IgG, respectively. The patient ID and the serogroup for which the patient tested positive are indicated above the graph. Only the results for patients with positive serology for any of the serogroups analyzed are shown.
