# Supplementary material for: Serology as an early diagnostic tool in pediatric patients with Shiga toxin-producing Escherichia coli-associated hemolytic uremic syndrome: a post hoc analysis of a phase 2 clinical trial
Source: J Clin Microbiol. 2026 Feb 27;64(4):e01415-25. doi: 10.1128/jcm.01415-25 (PMC13059726; doi:10.1128/jcm.01415-25)
Supplement: Table S1 — Diagnostic methodologies used in each of the centers that participated in the study. [file jcm.01415-25-s0002.pdf]

Table S1. Diagnostic methodologies used in each of the Centers that participated in the study.

Center	STEC diagnosis methods in stool samples
AR01	Conventional-PCR / Culture
AR02	Conventional-PCR / FilmArray/ Culture
AR04	RT-PCR / FilmArray/ Culture
AR05	FilmArray
AR06	RT-PCR / Culture
AR08	Conventional-PCR / FFStx / Culture
AR10	FFStx
AR11	Conventional-PCR / Culture
AR12	Conventional-PCR / Culture
AR13	FilmArray
AR15	EIA / Conventional-PCR / FFStx / Culture
AR16	FilmArray/ Culture
AR17	FFStx / Conventional-PCR / Culture
