# Supplementary material for: Serology as an early diagnostic tool in pediatric patients with Shiga toxin-producing Escherichia coli-associated hemolytic uremic syndrome: a post hoc analysis of a phase 2 clinical trial
Source: J Clin Microbiol. 2026 Feb 27;64(4):e01415-25. doi: 10.1128/jcm.01415-25 (PMC13059726; doi:10.1128/jcm.01415-25)
Supplement: Table S2 — Complete information and results of the samples analyzed in this study. [file jcm.01415-25-s0003.pdf]

Table S2. Complete information and results of the samples analyzed in this study.

											**CHEMSTRIP® *E. coli* O157/O145 (IgM) (g)**		**CHEMLIS® *E. coli* Glyco-iELISA (h)**				
**Patient ID**	**Age (years)**	**Diarrhea onset**	**Bloody diarrhea (BD) onset (a)**	**STEC-HUS diagnosis (b)**	**Timepoints of serum sampling (c)**	**Date of serum sample collection**	**Date of stool sample collection**	**DPOD (d)**	**Bacterial isolation and characterization (e)**	***stx*/Stx detection (detection method) (f)**	**O157 result**	**O145 result**	**IgM PR**	**IgM interpretation**	**IgG PR**	**IgG interpretation**	**Serogroup result (i)**
1	4	7/12/2022	8/12/2022	10/12/2022	T1	11/12/2022	2022/12/09	4	O145 stx2a/eae/ehxA	stx2 (PCR)	NEG	8	157,5	POS	106,5	POS	O145
					T3	13/12/2022		6			NEG	8	171,8	POS	110,4	POS	
					T7	17/12/2022		10			NEG	8	130,8	POS	82,0	POS	
					T28	6/1/2023		30			NEG	5	53,1	POS	38,3	IND	
2	1	10/4/2023	No BD	17/4/2023	T1	18/4/2023	2023/04/17	8	NEG	stx2 (PCR)	4	NEG	114,3	POS	105,2	POS	O157
					T3	20/4/2023		10			8	NEG	147,7	POS	141,2	POS	
					T7	24/4/2023		14			8	NEG	131,9	POS	143,5	POS	
					T28	15/5/2023		35			2	NEG	39,9	IND	114,7	POS	
3	3	22/11/2022	26/11/2022	29/11/2022	T1	30/11/2022	2022/11/30	8	NEG	NEG	8	NEG	179,1	POS	131,8	POS	O157
					T3	2/12/2022		10	(rectal swab)		9	NEG	174,4	POS	127,3	POS	
					T7	6/12/2022		14			9	NEG	151,2	POS	131,5	POS	
					T28	27/12/2022		35			4	NEG	72,8	POS	142,4	POS	
4	2	26/11/2022	No BD	30/11/2022	T1	1/12/2022	2022/12/01	5	O145 stx2a/eae/ehxA	stx2 (PCR)	NEG	7	181,2	POS	110,3	POS	O145
					T3	3/12/2022		7	(rectal swab)		NEG	5	155,0	POS	99,1	POS	
					T7	7/12/2022		11			NEG	7	173,4	POS	102,1	POS	
					T28	28/12/2022		32			NEG	4	60,1	POS	49,7	POS	
5	2	30/11/2022	30/11/2022	3/12/2022	T1	5/12/2022	2022/12/05	5	O157 stx2a_c/eae/exhA	stx2 (PCR)	9	NEG	171,3	POS	146,1	POS	O157
					T3	7/12/2022		7			8	NEG	161,5	POS	140,2	POS	
					T7	10/12/2022		10			6	NEG	83,7	POS	144,0	POS	
					T28	31/12/2022		31			3	NEG	62,6	POS	141,5	POS	
6	1	30/11/2022	4/12/2022	5/12/2022	T1	6/12/2022	2022/12/05	6	O157 stx2a_c/eae/exhA	stx2 (PCR)	3	NEG	89,8	POS	96,1	POS	O157
					T3	8/12/2022		8			NS	NS	167,3	POS	198,4	POS	
					T7	12/12/2022		12			7	NEG	165,2	POS	127,2	POS	
					T28	2/1/2023		33			3	NEG	92,0	POS	142,4	POS	

7	2	13/12/2022	16/12/2022	19/12/2022	T1	20/12/2022	2022/12/19	7	NEG	stx (FilmArray)	6	NEG	120,0	POS	102,8	POS	O157
					T3	22/12/2022		9			8	NEG	157,3	POS	147,4	POS	
					T7	26/12/2022		13			1	NEG	24,2	IND	87,1	POS	
					T28	16/1/2023		34			1	NEG	18,0	NEG	70,7	POS	
8	2	31/12/2022	1/1/2023	4/1/2023	T1	5/1/2023	2023/01/05	5	NEG	stx2 (PCR)	6	NEG	166,9	POS	150,3	POS	O157
					T3	7/1/2023		7			8	NEG	183,9	POS	139,2	POS	
					T7	11/1/2023		11			9	NEG	168,0	POS	135,3	POS	
					T28	1/2/2023		32			3	NEG	108,3	POS	143,5	POS	
9	1	4/1/2023	6/1/2023	10/1/2023	T1	11/1/2023	2023/01/11	7	NEG	stx2 (PCR)	4	NEG	109,9	POS	140,2	POS	O157
					T3	13/1/2023		9	(rectal swab)		5	NEG	100,6	POS	137,6	POS	
					T7	17/1/2023		13			3	NEG	69,8	POS	141,5	POS	
					T28	7/2/2023		34			1	NEG	18,2	NEG	146,6	POS	
10	1	10/1/2023	10/1/2023	13/1/2023	T1	14/1/2023	2023/01/13	4	NEG	stx2 (PCR)	NEG	10	277,9	POS	160,1	POS	O145
					T3	16/1/2023		6			NEG	9	264,7	POS	157,4	POS	
					T7	20/1/2023		10			NEG	8	244,7	POS	159,0	POS	
					T28	10/2/2023		31			NEG	2	146,6	POS	169,6	POS	
11	5	11/2/2023	11/2/2023	20/2/2023	T1	20/2/2023	2023/02/20	9	NEG	NEG	4	NEG	115,9	POS	142,4	POS	O157
					T3	22/2/2023		11	(rectal swab)		5	NEG	98,7	POS	139,5	POS	
					T7	27/2/2023		16			3	NEG	81,7	POS	133,9	POS	
					T28	20/3/2023		37			3	NEG	60,9	POS	125,4	POS	
12	6	21/2/2023	24/2/2023	1/3/2023	T1	2/3/2023	2023/03/02	9	NEG	stx1/stx2 (PCR)	2	1	83,2	POS	144,7	POS	O157
					T3	4/3/2023		11			2	NEG	69,7	POS	146,7	POS	
					T7	8/3/2023		15			1	NEG	55,4	POS	142,1	POS	
					T28	29/3/2023		36			1	NEG	42,6	IND	84,5	POS	
13	1	24/3/2023	No BD	30/3/2023	T1	31/3/2023	2023/03/29	7	NEG	stx2 (PCR)	3	NEG	80,4	POS	90,4	POS	O157
					T3	2/4/2023		9			3	NEG	69,6	POS	94,0	POS	
					T7	6/4/2023		13			3	NEG	68,6	POS	107,2	POS	
					T28	27/4/2023		34			1	NEG	22,7	NEG	58,7	IND	
14	1	6/4/2023	7/4/2023	9/4/2023	T1	10/4/2023	2023/04/06	4	NEG	stx2 (PCR)	9	NEG	181,2	POS	146,2	POS	O157
					T3	12/4/2023		6			9	NEG	180,1	POS	147,5	POS	
					T7	16/4/2023		10			9	NEG	168,0	POS	145,7	POS	
					T28	5/5/2023		29			5	NEG	106,4	POS	136,4	POS	
15	3	9/4/2023	10/4/2023	13/4/2023	T1	14/4/2023	2023/04/12	5	O157 stx2a/eae/exhA	stx2 (PCR)	9	NEG	179,9	POS	136,0	POS	O157

					T3	16/4/2023		7			8	NEG	177,8	POS	121,3	POS	
					T7	20/4/2023		11			10	NEG	169,9	POS	130,0	POS	
					T28	11/5/2023		32			4	NEG	140,3	POS	141,7	POS	
16	1	9/4/2023	10/4/2023	15/4/2023	T1	15/4/2023	2023/04/15	6	NEG	NEG	NEG	5	128,8	POS	81,5	POS	O145
					T3	17/4/2023		8	(rectal swab)		NEG	3	96,5	POS	68,4	POS	
					T7	21/4/2023		12			NEG	2	57,2	POS	64,1	POS	
					T28	12/5/2023		33			NEG	NEG	14,3	NEG	33,0	IND	
17	1	27/2/2023	27/2/2023	2/3/2023	T1	3/3/2023	2023/03/02	4	NEG	NEG	NEG	NEG					NEG
					T3	5/3/2023		6			NEG	NEG					
					T7	9/3/2023		10			NEG	NEG					
					T28	30/3/2023		31			NEG	NEG					
18	1	3/3/2023	No BD	7/3/2023	T1	8/3/2023	2023/03/08	5	O157 stx2a/eae/exhA	stx2 (PCR)	4	NEG	74,3	POS	131,5	POS	O157
					T3	10/3/2023		7			5	NEG	79,9	POS	144,1	POS	
					T7	14/3/2023		11			3	NEG	51,3	POS	143,5	POS	
					T28	4/4/2023		32			1	NEG	23,4	NEG	124,3	POS	
19	2	5/12/2022	7/12/2022	11/12/2022	T1	12/12/2022	2022/12/19	7	NEG	stx (FilmArray)	NEG	4	114,8	POS	66,8	POS	O145
					T3	14/12/2022		9			NEG	4	80,9	POS	48,3	POS	
					T7	19/12/2022		14			NEG	2	84,9	POS	58,8	POS	
					T28	6/1/2023		32			NEG	1	33,7	IND	77,6	POS	
20	4	16/12/2022	18/12/2022	22/12/2022	T1	22/12/2022	2022/12/20	6	NEG	stx (FilmArray)	9	NEG	181,6	POS	147,5	POS	O157
					T3	24/12/2022		8			9	NEG	182,0	POS	147,2	POS	
					T7	28/12/2022		12			9	NEG	176,5	POS	145,8	POS	
					T28	20/1/2023		35			8	NEG	154,3	POS	144,3	POS	
21	7	7/10/2022	8/10/2022	9/10/2022	T1	10/10/2022	2022/10/09	3	NEG	ND	NEG	NEG					NEG
					T3	12/10/2022		5			NEG	NEG					
					T7	17/10/2022		10			NS	NS					
					T28	7/11/2022		31			NEG	NEG					
22	8	22/12/2022	24/12/2022	25/12/2022	T1	26/12/2022	2022/12/26	4	NEG	stx2 (PCR)	NEG	NEG					NEG
					T3	28/12/2022		6	(rectal swab)		NEG	NEG					
					T7	2/1/2023		11			NEG	NEG					
					T28	23/1/2023		32			NEG	NEG					
23	3	28/12/2022	28/12/2022	3/1/2023	T1	3/1/2023	2023/01/03	6	NEG	ND	3	NEG	145,7	POS	137,9	POS	O157
					T3	5/1/2023		8			3	NEG	146,3	POS	141,8	POS	

					T7	10/1/2023		13			3	NEG	119,7	POS	132,2	POS	
					T28	31/1/2023		34			2	NEG	82,0	POS	109,7	POS	
24	5	12/1/2023	No BD	18/1/2023	T1	18/1/2023	2023/01/18	6	NEG	stx2 (PCR)	NEG	6	260,4	POS	170,7	POS	O145
					T3	20/1/2023		8			NEG	6	252,7	POS	171,3	POS	
					T7	25/1/2023		13			NEG	5	198,1	POS	170,0	POS	
					T28	14/2/2023		33			NEG	4	100,3	POS	189,5	POS	
25	5	22/12/2022	26/12/2022	1/1/2023	T1	2/1/2023	2022/12/26	11	O145<br>stx2a/eae/ehxA	stx2 (PCR)	NEG	3	114,1	POS	167,3	POS	O145
					T3	4/1/2023		13			NEG	2	90,9	POS	163,3	POS	
					T7	9/1/2023		18			NEG	2	77,5	POS	161,9	POS	
					T28	30/1/2023		39			1	1	41,8	IND	165,4	POS	
26	1	15/1/2023	15/1/2023	18/1/2023	T1	19/1/2023	2023/01/16	4	O157<br>stx2a_c/eae/exhA	stx2 (PCR)	4	NEG	107,7	POS	107,1	POS	O157
					T3	21/1/2023		6			9	NEG	181,5	POS	145,6	POS	
					T7	25/1/2023		10			9	NEG	178,7	POS	141,4	POS	
					T28	15/2/2023		31			5	NEG	144,8	POS	144,9	POS	
27	2	17/1/2023	No BD	22/1/2023	T1	23/1/2023	2023/01/22	6	NEG	stx2 (PCR)	NEG	6	201,9	POS	142,8	POS	O145
					T3	25/1/2023		8			NEG	7	185,2	POS	142,4	POS	
					T7	29/1/2023		12			NEG	7	102,6	POS	83,4	POS	
					T28	17/2/2023		31			NEG	2	51,3	POS	59,8	POS	
28	1	11/3/2023	11/3/2023	14/3/2023	T1	15/3/2023	2023/03/14	4	NEG	NEG	8	NEG	108,8	POS	144,7	POS	O157
					T3	16/3/2023		5			7	NEG	105,2	POS	143,5	POS	
					T7	21/3/2023		10			6	NEG	35,9	IND	142,8	POS	
					T28	11/4/2023		31			2	NEG	22,7	NEG	144,0	POS	
29	1	22/3/2023	No BD	30/3/2023	T1	30/3/2023	2023/03/30	8	NEG	ND	NEG	3	40,1	IND	28,4	IND	O145<br>(only by<br>CHEMSTRIP)
					T3	1/4/2023		10			NEG	2	40,3	IND	46,0	POS	
					T7	5/4/2023		14			NEG	2	27,1	IND	50,2	POS	
					T28	26/4/2023		35			NEG	1	10,3	NEG	33,1	IND	
30	1	26/4/2023	26/4/2023	30/4/2023	T1	30/4/2023	2023/05/02	4	NEG	stx (FFStx)	6	NEG	166,1	POS	143,9	POS	O157
					T3	2/5/2023		6			8	NEG	171,2	POS	138,1	POS	
					T7	8/5/2023		12			8	NEG	158,7	POS	142,1	POS	
					T28	29/5/2023		33			5	NEG	113,3	POS	141,1	POS	
31	4	10/12/2022	16/12/2022	20/12/2022	T1	20/12/2022	2022/12/26	10	NEG	NEG	NEG	6	95,9	POS	69,8	POS	O145
					T3	22/12/2022		12			NEG	7	145,9	POS	103,3	POS	
					T7	27/12/2022		17			NEG	6	69,5	POS	94,5	POS	

					T28	17/1/2023		38			NEG	2	24,8	IND	78,2	POS	
32	2	10/1/2023	11/1/2023	15/1/2023	T1	16/1/2023	2023/01/24	6	NEG	stx1/stx2 (PCR)	10	NEG	180,7	POS	140,3	POS	O157
					T3	18/1/2023		8			10	NEG	181,3	POS	140,5	POS	
					T7	23/1/2023		13			10	NEG	178,0	POS	138,4	POS	
					T28	13/2/2023		34			9	NEG	162,6	POS	137,9	POS	
33	2	10/4/2023	13/4/2023	16/4/2023	T1	17/4/2023	2023/04/17	7	NEG	stx2 (PCR)	NEG	5	100,0	POS	86,5	POS	O145
					T3	19/4/2023		9			NEG	5	131,1	POS	124,7	POS	
					T7	24/4/2023		14			NEG	5	81,4	POS	114,8	POS	
					T28	12/5/2023		32			NEG	2	43,4	IND	126,5	POS	
34	1	8/12/2022	13/12/2022	15/12/2022	T1	15/12/2022	2022/12/13	7	NEG	stx (FilmArray)	8	NEG	175,4	POS	147,2	POS	O157
					T3	17/12/2022		9			9	NEG	175,3	POS	148,1	POS	
					T7	22/12/2022		14			8	NEG	160,1	POS	146,0	POS	
					T28	11/1/2023		34			8	NEG	131,3	POS	141,6	POS	
35	3	31/1/2023	No BD	2/2/2023	T1	3/2/2023	2023/02/02	3	NEG	stx (FilmArray)	7	NEG	151,4	POS	141,3	POS	O157
					T3	5/2/2023		5			8	NEG	171,0	POS	126,9	POS	
					T7	9/2/2023		9			9	NEG	154,1	POS	129,6	POS	
					T28	2/3/2023		30			4	NEG	112,9	POS	136,4	POS	
36	3	9/3/2023	No BD	17/3/2023	T1	17/3/2023	2023/03/21	8	NEG	stx (FilmArray)	6	NEG	161,3	POS	137,0	POS	O157
					T3	19/3/2023		10			6	NEG	155,3	POS	140,3	POS	
					T7	23/3/2023		14			6	NEG	126,3	POS	143,7	POS	
					T28	14/4/2023		36			2	NEG	59,4	POS	148,0	POS	
37	1	1/12/2022	6/12/2022	6/12/2022	T1	6/12/2022	2022/12/06	5	NEG	stx2 (PCR)	NEG	NEG					NEG
					T3	8/12/2022		7			NEG	NEG					
					T7	14/12/2022		13			SM	SM					
					T28	4/1/2023		34			NEG	NEG					
38	1	8/12/2022	8/12/2022	10/12/2022	T1	12/12/2022	2022/12/12	4	NEG	stx2 (PCR)	8	NEG	168,5	POS	142,4	POS	O157
					T3	14/12/2022		6			9	NEG	178,5	POS	137,6	POS	
					T7	16/12/2022		8			8	NEG	177,6	POS	135,7	POS	
					T28	6/1/2023		29			8	NEG	157,1	POS	141,5	POS	
39	3	30/12/2022	No BD	2/1/2023	T1	2/1/2023	2023/01/02	3	NEG	NEG	4	NEG	161,8	POS	141,1	POS	O157
					T3	4/1/2023		5			5	NEG	153,8	POS	137,8	POS	
					T7	9/1/2023		10			3	NEG	140,1	POS	133,7	POS	
					T28	31/1/2023		32			1	NEG	82,0	POS	112,4	POS	

40	1	4/1/2023	4/1/2023	5/1/2023	T1	6/1/2023	2023/01/06	2	O157 eae/exhA	stx2 (PCR)	3	NEG	57,8	POS	124,7	POS	O157
					T3	8/1/2023		4			5	NEG	125,5	POS	139,3	POS	
					T7	13/1/2023		9			5	NEG	80,3	POS	141,1	POS	
					T28	3/2/2023		30			2	NEG	24,4	IND	141,7	POS	
41	4	20/1/2023	22/1/2023	30/1/2023	T1	30/1/2023	2023/01/24	10	NEG	ND	1	NEG	58,3	POS	132,6	POS	O157
					T3	1/2/2023		12			2	NEG	54,6	POS	135,2	POS	
					T7	7/2/2023		18			1	NEG	44,1	POS	141,7	POS	
					T28	28/2/2023		39			1	NEG	68,5	POS	145,9	POS	
42	3	30/1/2023	No BD	1/2/2023	T1	1/2/2023	2023/02/03	2	NEG	NEG	NEG	NEG	70,8	POS	206,2	POS	O111
					T3	3/2/2023		4			NEG	NEG	83,7	POS	277,4	POS	
					T7	7/2/2023		8			NEG	NEG	74,0	POS	334,4	POS	
					T28	2/3/2023		31			NEG	NEG	31,0	NEG	310,5	POS	
43	1	3/2/2023	3/2/2023	7/2/2023	T1	8/2/2023	2023/02/10	5	NEG	NEG	NEG	8	207,7	POS	119,2	POS	O145
					T3	10/2/2023		7			NEG	9	260,1	POS	160,1	POS	
					T7	14/2/2023		11			NEG	6	220,2	POS	147,9	POS	
					T28	9/3/2023		34			NEG	3	68,2	POS	68,2	POS	
44	1	7/2/2023	7/2/2023	15/2/2023	T1	15/2/2023	2023/02/17	8	NEG	NEG	9	NEG	194,9	POS	127,9	POS	O157
					T3	17/2/2023		10			8	NEG	193,7	POS	129,6	POS	
					T7	22/2/2023		15			9	NEG	182,3	POS	129,0	POS	
					T28	17/3/2023		38			6	NEG	112,8	POS	140,5	POS	
45	4	20/2/2023	21/2/2023	23/2/2023	T1	24/2/2023	2023/02/23	4	NEG	stx2 (PCR)	NEG	NEG	420,0	POS	169,4	POS	O121
					T3	26/2/2023		6			NEG	NEG	460,3	POS	185,0	POS	
					T7	2/3/2023		10			NEG	NEG	462,7	POS	188,3	POS	
					T28	21/3/2023		29			NEG	NEG	434,6	POS	188,9	POS	
46	3	2/3/2023	6/3/2023	8/3/2023	T1	9/3/2023	2023/03/09	7	NEG	stx2 (PCR)	NEG	4	222,9	POS	156,8	POS	O145
					T3	11/3/2023		9			NEG	2	194,3	POS	151,7	POS	
					T7	15/3/2023		13			NEG	3	172,0	POS	152,5	POS	
					T28	5/4/2023		34			NEG	2	54,3	POS	154,9	POS	
47	7	26/3/2023	26/3/2023	31/3/2023	T1	31/3/2023	2023/03/31	5	NEG	NEG	NEG	NEG	100	POS	246	POS	O111
					T3	2/4/2023		7			NEG	NEG	88	POS	218	POS	
					T7	5/4/2023		10			NEG	NEG	67	POS	157	POS	
					T28	27/4/2023		32			1	NEG	29	NEG	50	POS	
48	4	1/12/2022	3/12/2022	8/12/2022	T1	8/12/2022	2022/12/15	7	NEG	ND	8	NEG	167,1	POS	138,0	POS	O157
					T3	10/12/2022		9			7	NEG	141,3	POS	130,0	POS	

					T7	15/12/2022		14			6	NEG	122,7	POS	124,1	POS	
					T28	5/1/2023		35			4	NEG	41,3	IND	74,3	POS	
49	2	28/12/2022	2/1/2023	2/1/2023	T1	3/1/2023	2023/01/02	6	NEG	stx2 (PCR)	9	NEG	191,9	POS	146,4	POS	O157
					T3	5/1/2023		8			10	NEG	190,7	POS	129,0	POS	
					T7	10/1/2023		13			9	NEG	188,2	POS	130,0	POS	
					T28	30/1/2023		33			8	NEG	183,8	POS	123,5	POS	
50	3	7/1/2023	8/1/2023	9/1/2023	T1	10/1/2023	2023/01/10	3	NEG	stx (FilmArray)	NEG	NEG					NEG
					T3	12/1/2023		5			NEG	NEG					
					T7	16/1/2023		9			NEG	NEG					
					T28	6/2/2023		30			NEG	NEG					
51	8	4/2/2023	5/2/2023	7/2/2023	T1	7/2/2023	2023/02/06	3	NEG	stx (FilmArray)	1	NEG	63,6	POS	37,4	NEG	O157
					T3	9/2/2023		5			3	NEG	110,6	POS	103,8	POS	
					T7	14/2/2023		10			4	NEG	136,0	POS	133,2	POS	
					T28	7/3/2023		31			3	NEG	107,7	POS	111,4	POS	
52	1	2/3/2023	2/3/2023	9/3/2023	T1	10/3/2023	2023/03/03	8	NEG	stx2 (PCR)	NEG	7	255,3	POS	166,6	POS	O145
					T3	12/3/2023		10			NEG	6	236,5	POS	155,4	POS	
					T7	17/3/2023		15			NEG	5	179,0	POS	123,9	POS	
					T28	5/4/2023		34			NEG	1	48,2	POS	71,9	POS	
53	1	7/4/2023	7/4/2023	8/4/2023	T1	9/4/2023	2023/04/09	2	NEG	stx (FilmArray)	NEG	NEG					NEG
					T3	11/4/2023		4			NEG	NEG					
					T7	15/4/2023		8			NEG	NEG					
					T28	5/5/2023		28			NEG	NEG					
54	1	31/1/2023	31/1/2023	4/2/2023	T1	5/2/2023	2023/02/07	5	NEG	NEG	NEG	3	122,1	POS	77,0	POS	O145
					T3	7/2/2023		7	(rectal swab)		NEG	3	110,4	POS	67,6	POS	
					T7	11/2/2023		11			NEG	3	75,0	POS	54,0	POS	
					T28	3/3/2023		31			NEG	1	35,4	IND	67,0	POS	
55	1	2/3/2023	2/3/2023	7/3/2023	T1	8/3/2023	2023/03/07	6	NEG	NEG	8	NEG	181,9	POS	143,4	POS	O157
					T3	10/3/2023		8			7	NEG	182,9	POS	140,3	POS	
					T7	14/3/2023		12			6	NEG	173,2	POS	143,9	POS	
					T28	4/4/2023		33			5	NEG	85,5	POS	124,6	POS	

[a] No BD, the patient did not have acute bloody diarrhea.

[b] STEC-HUS compatible diagnosis was defined as signs of kidney damage (serum creatinine above the Upper Limit of Normal -ULN- for age and sex, and/or hematuria - ≥5 red blood cells per field or ≥27 red blood cells/μl in urinary sediment-) and at least one of the following two criteria: presence of hemolysis (lactate dehydrogenase above ULN for age and sex and/or schistocytes

in peripheral blood spread) and/or platelet consumption (platelet count <150 x10^3/µl in peripheral blood and/or ≥50% decrease in peripheral blood platelet count from baseline or within the previous 24 hours).

[c] Four serum samples were collected for each patient: T1; 12 to 24 h after STEC-HUS diagnosis and before dose 1 administration of INM004; T3, 48 h after T1 and 24 h after dose 2 administration of INM004; T7, 7 days after STEC-HUS diagnosis; and T28, 28 days after STEC-HUS diagnosis

[d] DPOD, days post-onset of diarrhea, calculated as the days elapsed between the onset of diarrhea and serum sample collection at T1.

[e] Stool culture from 49 fresh stools and 6 rectal swabs. NEG, negative result.

[f] *stx*/Stx detection, FFStx Shiga toxin detection and/or *stx* gene detection by PCR. NEG, negative result. ND, not done.

[g] T1, T3, T7 and T28 serum samples were analyzed by the CHEMSTRIP® *E. coli* O157/O145 (Chemtest Argentina S. A.) immunochromatographic test. Results are expressed as the color intensity (CI) of the TL determined by visual inspection and registered based on a 0-10 color grading scale. Interpretation of the result: CI = 0, negative (NEG); CI > 0, positive (POS). NS, no sample available.

[h] CHEMLIS® *E. coli* Glyco-iELISA (Chemtest Argentina S. A.) analysis of T1, T3, T7 and T28 serum samples. Results were expressed as the percentage of reactivity (PR) with respect to the corresponding positive control. Only the PR values for the Glyco-iELISA that tested positive are shown. Empty cells indicate a negative result for all serogroups tested.

CHEMLIS® *E. coli* O157 Glyco-iELISA interpretation of the results. IgM: PR ≥ 43%, positive (POS); 24% < PR < 43%, indeterminate (IND); PR ≤ 24%, negative (NEG). IgG: PR ≥ 60%, positive (POS); 47% < PR < 60%, indeterminate (IND); PR ≤ 47%, negative (NEG).

CHEMLIS® *E. coli* O145 Glyco-iELISA interpretation of the results. IgM: PR ≥ 46%, positive; 18% < PR < 46%, indeterminate; PR ≤ 18%, negative. IgG: PR ≥ 41%, positive; 23% < PR < 41%, indeterminate; PR ≤ 23%, negative,

CHEMLIS® *E. coli* O121 Glyco-iELISA, interpretation of the results. IgM: PR ≥ 40%, positive; 25% < PR < 40%, indeterminate; PR ≤ 25%, negative, IgG: PR ≥ 44%, positive; PR < 44%, negative.

CHEMLIS® *E. coli* O111 Glyco-iELISA, interpretation of the results. IgM: PR ≥ 35%, positive; PR ≤ 35%, negative. IgG, PR ≥ 35%, positive; PR < 35%, negative.

[i] *E. coli* serogroup identified by CHEMLIS® *E. coli* Glyco-iELISAs and/or CHEMSTRIP® *E. coli* O157/O145.
