# Supplementary material for: Serology as an early diagnostic tool in pediatric patients with Shiga toxin-producing Escherichia coli-associated hemolytic uremic syndrome: a post hoc analysis of a phase 2 clinical trial
Source: J Clin Microbiol. 2026 Feb 27;64(4):e01415-25. doi: 10.1128/jcm.01415-25 (PMC13059726; doi:10.1128/jcm.01415-25)
Supplement: Table S3 — Complete information and results of T1 samples sorted according to the days post-onset of diarrhea (DPOD). [file jcm.01415-25-s0004.pdf]

Table S3. Complete information and results of T1 samples sorted according to the days post-onset of diarrhea (DPOD).

									CHEMSTRIP® *E. coli* O157/O145 (IgM) (g)		CHEMLIS® *E. coli* Glyco-iELISA (h)				
**Patient ID**	**Age (years)**	**Diarrhea onset**	**Bloody diarrhea (BD) onset (a)**	**STEC-HUS diagnosis (b)**	**Timepoints of serum sampling (c)**	**DPOD (d)**	**Bacterial isolation and characterization (e)**	***stx*/Stx detection (detection method) (f)**	**O157 result**	**O145 result**	**IgM PR**	**IgM interpretation**	**IgG PR**	**IgG interpretation**	**Serogroup result (i)**
40	1	4/1/2023	4/1/2023	5/1/2023	T1	2	O157 eae/exhA	stx2 (PCR)	3	NEG	57,8	POS	124,7	POS	O157
42	3	30/1/2023	No BD	1/2/2023	T1	2	NEG	NEG	NEG	NEG	70,8	POS	206,2	POS	O111
53	1	7/4/2023	7/4/2023	8/4/2023	T1	2	NEG	stx (FilmArray)	NEG	NEG					NEG
21	7	7/10/2022	8/10/2022	9/10/2022	T1	3	NEG	ND	NEG	NEG					NEG
35	3	31/1/2023	No BD	2/2/2023	T1	3	NEG	stx (FilmArray)	7	NEG	151,4	POS	141,3	POS	O157
39	3	30/12/2022	No BD	2/1/2023	T1	3	NEG	NEG	4	NEG	161,8	POS	141,1	POS	O157
50	3	7/1/2023	8/1/2023	9/1/2023	T1	3	NEG	stx (FilmArray)	NEG	NEG					NEG
51	8	4/2/2023	5/2/2023	7/2/2023	T1	3	NEG	stx (FilmArray)	1	NEG	63,6	POS	37,4	NEG	O157
1	4	7/12/2022	8/12/2022	10/12/2022	T1	4	O145 stx2a/eae/ehxA	stx2 (PCR)	NEG	8	157,5	POS	106,5	POS	O145
10	1	10/1/2023	10/1/2023	13/1/2023	T1	4	NEG	stx2 (PCR)	NEG	10	277,9	POS	160,1	POS	O145
14	1	6/4/2023	7/4/2023	9/4/2023	T1	4	NEG	stx2 (PCR)	9	NEG	181,2	POS	146,2	POS	O157
17	1	27/2/2023	27/2/2023	2/3/2023	T1	4	NEG	NEG	NEG	NEG					NEG
22	8	22/12/2022	24/12/2022	25/12/2022	T1	4	NEG	stx2 (PCR)	NEG	NEG					NEG
26	1	15/1/2023	15/1/2023	18/1/2023	T1	4	O157 stx2a_c/eae/exhA	stx2 (PCR)	4	NEG	107,7	POS	107,1	POS	O157
28	1	11/3/2023	11/3/2023	14/3/2023	T1	4	NEG	NEG	8	NEG	108,8	POS	144,7	POS	O157
30	1	26/4/2023	26/4/2023	30/4/2023	T1	4	NEG	stx (FFStx)	6	NEG	166,1	POS	143,9	POS	O157
38	1	8/12/2022	8/12/2022	10/12/2022	T1	4	NEG	stx2 (PCR)	8	NEG	168,5	POS	142,4	POS	O157
45	4	20/2/2023	21/2/2023	23/2/2023	T1	4	NEG	stx2 (PCR)	NEG	NEG	420,0	POS	169,4	POS	O121
4	2	26/11/2022	No BD	30/11/2022	T1	5	O145 stx2a/eae/ehxA	stx2 (PCR)	NEG	7	181,2	POS	110,3	POS	O145
5	2	30/11/2022	30/11/2022	3/12/2022	T1	5	O157 stx2a_c/eae/exhA	stx2 (PCR)	9	NEG	171,3	POS	146,1	POS	O157
8	2	31/12/2022	1/1/2023	4/1/2023	T1	5	NEG	stx2 (PCR)	6	NEG	166,9	POS	150,3	POS	O157
15	3	9/4/2023	10/4/2023	13/4/2023	T1	5	O157 stx2a/eae/exhA	stx2 (PCR)	9	NEG	179,9	POS	136,0	POS	O157
18	1	3/3/2023	No BD	7/3/2023	T1	5	O157 stx2a/eae/exhA	stx2 (PCR)	4	NEG	74,3	POS	131,5	POS	O157
37	1	1/12/2022	6/12/2022	6/12/2022	T1	5	NEG	stx2 (PCR)	NEG	NEG					NEG
43	1	3/2/2023	3/2/2023	7/2/2023	T1	5	NEG	NEG	NEG	8	207,7	POS	119,2	POS	O145

47	7	26/3/2023	26/3/2023	31/3/2023	T1	5	NEG	NEG	NEG	NEG	100	POS	246	POS	O111
54	1	31/1/2023	31/1/2023	4/2/2023	T1	5	NEG	NEG	NEG	3	122,1	POS	77,0	POS	O145
6	1	30/11/2022	4/12/2022	5/12/2022	T1	6	O157 stx2a_c/eae/exhA	stx2 (PCR)	3	NEG	89,8	POS	96,1	POS	O157
16	1	9/4/2023	10/4/2023	15/4/2023	T1	6	NEG	NEG	NEG	5	128,8	POS	81,5	POS	O145
20	4	16/12/2022	18/12/2022	22/12/2022	T1	6	NEG	stx (FilmArray)	9	NEG	181,6	POS	147,5	POS	O157
23	3	28/12/2022	28/12/2022	3/1/2023	T1	6	NEG	ND	3	NEG	145,7	POS	137,9	POS	O157
24	5	12/1/2023	No BD	18/1/2023	T1	6	NEG	stx2 (PCR)	NEG	6	260,4	POS	170,7	POS	O145
27	2	17/1/2023	No BD	22/1/2023	T1	6	NEG	stx2 (PCR)	NEG	6	201,9	POS	142,8	POS	O145
32	2	10/1/2023	11/1/2023	15/1/2023	T1	6	NEG	stx1/stx2 (PCR)	10	NEG	180,7	POS	140,3	POS	O157
49	2	28/12/2022	2/1/2023	2/1/2023	T1	6	NEG	stx2 (PCR)	9	NEG	191,9	POS	146,4	POS	O157
55	1	2/3/2023	2/3/2023	7/3/2023	T1	6	NEG	NEG	8	NEG	181,9	POS	143,4	POS	O157
7	2	13/12/2022	16/12/2022	19/12/2022	T1	7	NEG	stx (FilmArray)	6	NEG	120,0	POS	102,8	POS	O157
9	1	4/1/2023	6/1/2023	10/1/2023	T1	7	NEG	stx2 (PCR)	4	NEG	109,9	POS	140,2	POS	O157
13	1	24/3/2023	No BD	30/3/2023	T1	7	NEG	stx2 (PCR)	3	NEG	80,4	POS	90,4	POS	O157
19	2	5/12/2022	7/12/2022	11/12/2022	T1	7	NEG	stx (FilmArray)	NEG	4	114,8	POS	66,8	POS	O145
33	2	10/4/2023	13/4/2023	16/4/2023	T1	7	NEG	stx2 (PCR)	NEG	5	100,0	POS	86,5	POS	O145
34	1	8/12/2022	13/12/2022	15/12/2022	T1	7	NEG	stx (FilmArray)	8	NEG	175,4	POS	147,2	POS	O157
46	3	2/3/2023	6/3/2023	8/3/2023	T1	7	NEG	stx2 (PCR)	NEG	4	222,9	POS	156,8	POS	O145
48	4	1/12/2022	3/12/2022	8/12/2022	T1	7	NEG	ND	8	NEG	167,1	POS	138,0	POS	O157
2	1	10/4/2023	No BD	17/4/2023	T1	8	NEG	stx2 (PCR)	4	NEG	114,3	POS	105,2	POS	O157
3	3	22/11/2022	26/11/2022	29/11/2022	T1	8	NEG	NEG	8	NEG	179,1	POS	131,8	POS	O157
29	1	22/3/2023	No BD	30/3/2023	T1	8	NEG	ND	NEG	3	40,1	IND	28,4	IND	O145
36	3	9/3/2023	No BD	17/3/2023	T1	8	NEG	stx (FilmArray)	6	NEG	161,3	POS	137,0	POS	O157
44	1	7/2/2023	7/2/2023	15/2/2023	T1	8	NEG	NEG	9	NEG	194,9	POS	127,9	POS	O157
52	1	2/3/2023	2/3/2023	9/3/2023	T1	8	NEG	stx2 (PCR)	NEG	7	255,3	POS	166,6	POS	O145
11	5	11/2/2023	11/2/2023	20/2/2023	T1	9	NEG	NEG	4	NEG	115,9	POS	142,4	POS	O157
12	6	21/2/2023	24/2/2023	1/3/2023	T1	9	NEG	stx1/stx2 (PCR)	2	1	83,2	POS	144,7	POS	O157
31	4	10/12/2022	16/12/2022	20/12/2022	T1	10	NEG	NEG	NEG	6	95,9	POS	69,8	POS	O145
41	4	20/1/2023	22/1/2023	30/1/2023	T1	10	NEG	ND	1	NEG	58,3	POS	132,6	POS	O157
25	5	22/12/2022	26/12/2022	1/1/2023	T1	11	O145 stx2a/eae/ehxA	stx2 (PCR)	NEG	3	114,1	POS	167,3	POS	O145

[a] No BD, the patient did not have acute bloody diarrhea.

[b] STEC-HUS compatible diagnosis was defined as signs of kidney damage (serum creatinine above the Upper Limit of Normal -ULN- for age and sex, and/or hematuria - ≥5 red blood cells per field or ≥27 red blood cells/µl in urinary sediment-) and at least one of the following two criteria: presence of hemolysis (lactate dehydrogenase above ULN for age and sex and/or schistocytes in peripheral blood spread) and/or platelet consumption (platelet count <150 x10^3/µl in peripheral blood and/or ≥50% decrease in peripheral blood platelet count from baseline or within the previous 24 hours).

[c] T1; the saerum sampler as collected 12 to 24 h after STEC-HUS diagnosis and before dose 1 administration of INM004.

[d] DPOD, days post-onset of diarrhea, calculated as the days elapsed between the onset of diarrhea and serum sample collection at T1.

[e] Stool culture from 49 fresh stools and 6 rectal swabs. NEG, negative result.

[f] *stx*/Stx detection, FFStx Shiga toxin detection and/or *stx* gene detection by PCR. NEG, negative result. ND, not done.

[g] Analysis by the CHEMSTRIP® *E. coli* O157/O145 (Chemtest Argentina S. A.) immunochromatographic test. Results are expressed as the color intensity (CI) of the TL determined by visual inspection and registered based on a 0-10 color grading scale. Interpretation of the result: CI = 0, negative (NEG); CI > 0, positive (POS). NS, no sample available.

[h] CHEMLIS® *E. coli* Glyco-iELISA (Chemtest Argentina S. A.) analysis. Results were expressed as the percentage of reactivity (PR) with respect to the corresponding positive control. Only the PR values for the Glyco-iELISA that tested positive are shown. Empty cells indicate a negative result for all serogroups tested.

CHEMLIS® *E. coli* O157 Glyco-iELISA interpretation of the results. IgM: PR ≥ 43%, positive (POS); 24% < PR < 43%, indeterminate (IND); PR ≤ 24%, negative (NEG). IgG: PR ≥ 60%, positive (POS); 47% < PR < 60%, indeterminate (IND); PR ≤ 47%, negative (NEG).

CHEMLIS® *E. coli* O145 Glyco-iELISA interpretation of the results. IgM: PR ≥ 46%, positive; 18% < PR < 46%, indeterminate; PR ≤ 18%, negative. IgG: PR ≥ 41%, positive; 23% < PR < 41%, indeterminate; PR ≤ 23%, negative,

CHEMLIS® *E. coli* O121 Glyco-iELISA, interpretation of the results. IgM: PR ≥ 40%, positive; 25% < PR < 40%, indeterminate; PR ≤ 25%, negative, IgG: PR ≥ 44%, positive; PR < 44%, negative.

CHEMLIS® *E. coli* O111 Glyco-iELISA, interpretation of the results. IgM: PR ≥ 35%, positive; PR ≤ 35%, negative. IgG, PR ≥ 35%, positive; PR < 35%, negative.

[i] *E. coli* serogroup identified by CHEMLIS® *E. coli* Glyco-iELISAs and/or CHEMSTRIP® *E. coli* O157/O145.
